# Tissue-specific transcriptomic analysis uncovers potential roles of natural antisense transcripts in *Arabidopsis* heat stress response

**DOI:** 10.3389/fpls.2022.997967

**Published:** 2022-09-08

**Authors:** Jingjing Jin, Naohiko Ohama, Xiujing He, Hui-Wen Wu, Nam-Hai Chua

**Affiliations:** ^1^China Tobacco Gene Research Center, Zhengzhou Tobacco Research Institute of CNTC, Zhengzhou, China; ^2^Temasek Life Sciences Laboratory, National University of Singapore, Singapore, Singapore; ^3^West China Hospital, Sichuan University, Chengdu, China

**Keywords:** long non-coding RNA, natural antisense transcript, heat stress, epigenetics, small RNA

## Abstract

Natural antisense transcripts (NATs) are an important class of non-coding ribonucleic acids (RNAs) that have been shown to regulate gene expression. Using strand-specific RNA sequencing, 36,317 NAT pairs were identified, and 5,536 were specifically expressed under heat stress. We found distinct expression patterns between vegetative and reproductive tissues for both coding genes and genes encoding NATs. Genes for heat-responsive NATs are associated with relatively high levels of H3K4me3 and low levels of H3K27me2/3. On the other hand, small RNAs are significantly enriched in sequence overlapping regions of NAT pairs, and a large number of heat-responsive NATs pairs serve as potential precursors of nat-siRNAs. Collectively, our results suggest epigenetic modifications and small RNAs play important roles in the regulation of NAT expression, and highlight the potential significance of heat-inducible NATs.

## Introduction

Global climate change has tremendous influence on food production because extreme temperatures will seriously hinder and reduce crop productivity (Lobell et al., 2011; Teixeira et al., 2013; Sultan et al., 2019). One way to mitigate impact of high temperature on crop productivity is to improve high temperature tolerance of crops. As a first step toward this end, it is important to identify key pathways and regulators as possible targets for thermo-tolerance improvement by performing genome-wide analysis of plant responses under heat stress. In order to successfully survive at high temperature, plants have evolved various physiological and molecular mechanisms to tolerate heat stress, most of which are conserved between different plant species. Heat stress can be induced by several mechanisms, such as calcium channel on the plasma membrane (Sajid et al., 2018), a mass of unfolded proteins in the endoplasmic reticulum and cytosol (Ding et al., 2020) and reactive oxygen species (ROS) (Suzuki and Mittler, 2006). These signals finally activate downstream genes including those encoding heat-shock transcription factors (HSF), multiprotein-bridging factor 1c, basic leucine zipper (bZIP) transcription factors and WRKYGQK motif-containing (WRKY) proteins (Ohama et al., 2017; Sajid et al., 2018; Zhao et al., 2020). Together, these factors control the expression of genes for heat shock proteins (HSPs) which are the most important regulators of heat stress response.

Natural antisense transcripts (NATs) are one important class of non-coding ribonucleic acids (RNAs), which are transcribed from strands opposite to sense transcripts of coding or non-coding genes. First identified in bacteria, NATs have since been discovered in both animals and plants. Genome-wide analysis in *Arabidopsis* (Wang et al., 2014), rice (Lu et al., 2012), wheat (Coram et al., 2009), *Brassica rapa* (Yu et al., 2013), and sugarcane (Lembke et al., 2012) show that NATs pairs are prevalent in both monocots and dicots. Wang et al. found 37,238 sense-antisense transcript pairs and 70% of the annotated RNAs are associated with antisense transcripts (Wang et al., 2014). Lu et al. identified 2,292 rice cis-NATs with detectable transcripts and obtained nat-siRNAs evidence among sequence overlapping regions of NATs (Lu et al., 2012). Xu et al. (2017) identified 1,769 NAT pairs exhibiting different sensitivity to drought stress. These studies suggest involvement of NAT pairs in responses to various abiotic and biotic stresses.

Various mechanisms have been used by NATs to regulate sense transcripts at the transcriptional or post-transcriptional level. The cold-induced *COOLAIR* transcription accelerates repression of FLOWERING LOCUS C (*FLC*) transcription via changes in histone marks (Heo and Sung, 2011). Similarly, we have demonstrated that the AG-incRNA4 can be associated with CURLY LEAF (*CLF*) to repress AGAMOUS (*AG*) expression in leaf tissues through H3K27me3-mediated repression (Wu et al., 2018). A cis-NATPHO1;2 can promote translation of its sense transcript HOSPHATE1;2 (*PHO1;2*) for phosphate homeostasis and plant fitness in rice (Jabnoune et al., 2013). Another rice cis-natural antisense lncRNA, TWISTED LEAF (*TL*) plays important role in maintaining leaf blade flattening (Liu et al., 2018). Overexpression of LAIR (LRK Antisense Intergenic RNA) increases rice grain yield and promotes the expression of several LRK genes (Wang et al., 2018). Interestingly, another study has demonstrated that NAT-398b/c could repress miR398 biogenesis and attenuate plant thermotolerance via a regulatory loop (Li et al., 2020).

Here, we investigated whether long non-coding RNA, especially NATs, may be specifically involved in heat response. We compared transcriptome (coding and non-coding) profiles between reproductive tissues (unopened buds, open flowers) and vegetative tissues (rosette leaf, shoots, and roots). Similar with previous results, we also discovered distinct heat stress response patterns between vegetative and reproductive tissues. Moreover, a large number of NAT genes were responsive to heat treatment and showed similar tissue-specific expression profiles as their cognate coding genes. Based on previously published epigenetic data, a strong association between heat-regulated NAT gene pairs and epigenetic markers were found. Moreover, smRNAs were significantly enriched in the overlapping region of NAT pairs and around 3,000 heat-responsive NAT pairs served as potential precursors of nat-siRNAs. Our study reveals a potential role for NAT pairs in nat-siRNA-mediated heat response.

## Materials and methods

### Plant materials

*Arabidopsis thaliana* ecotype Columbia (Col-0) was used as the wild type. The *hsfa1* quadruple mutant was described previously (Yoshida et al., 2011). Seeds were surface-sterilized and sown on Murashige and Skoog (MS) medium plates containing 1% sucrose and 0.8% agar. After stratification at 4°C for 2 days, MS plates were transferred to a growth chamber at 22°C with long day photoperiod (16-h light/8-h dark). To prepare soil-grown plant material, 1-week-old seedlings grown on MS plates were transferred to soil and incubated under the same conditions as MS plates. RNA samples were prepared from 14-day-old young seedlings grown on agar plates and 4-week-old soil-grown adult plants using RNeasy Plant Mini Kit (QIAGEN, Catalog number: 74904). After 0, 1, and 5 h of heat stress treatments at 37°C, young seedlings were dissected into shoot and root samples. Adult plants were dissected into rosette leaf (RL), unopened bud (Bud) and open flower (OF) samples. These samples included 2 reproductive tissues (OF and Bud) and 3 vegetative tissues (Shoot, Root, and RL).

### Annotation of long non-coding RNAs (lncRNAs)

Strand-specific lncRNA sequencing was conducted by Novogene Company according to standard protocols (Li et al., 2021). Clean data were mapped to the reference genome (TAIR10) using HIAST2 (Kim et al., 2015). The transcriptome dataset for each library was separately assembled by StringTie (Pertea et al., 2015). Then, gtf files for each library were combined into one with StringTie–merge. The assembled transcripts were compared with reference genome annotation (TAIR10). Transcripts with a sequence shorter than 200 nt were discarded. The transcripts were further filtered to remove those with sequence-overlap with tRNA, rRNA, sRNA, and miRNA in Rfam (Mistry et al., 2021) database. Coding potential of the remaining transcripts were measured by the CPC (Kong et al., 2007) program. The surviving transcripts were regarded as long intergenic non-coding RNA (lincRNA) candidates if they mapped to intergenic regions. Transcripts encoded by intronic deoxyribonucleic acid (DNA) sequences of known genes were considered as intronic RNA (incRNA) candidates. Transcripts were considered as NATs candidates if they were transcribed from the opposite strands of known genes. These transcripts were compared with lincRNA and NATs annotation from our previous studies (Liu et al., 2012; Wang et al., 2014). The expression values for lncRNAs were measured using TPM as previously described (Jin et al., 2021).

### Gene ontology enrichment analysis

GO enrichment analysis on target genes was performed on the KOBAS website (Bu et al., 2021). GO terms with a corrected *p*-value smaller than 0.05 were regarded as significantly enriched terms.

### Quantitative RT-PCR

One μg of total RNA was reverse-transcribed with qScript cDNA synthesis kit (Quanta bio, Catalog number: 95048-100). qPCR was performed using SsoAdvanced Universal SYBR Green Supermix (Bio-Rad, Catalog number: 1725274) on a CFX96 Real-Time PCR Detection System (Bio-Rad). Expression data were analyzed using CFX Manager (Bio-Rad). Expression levels were normalized to *Actin2* expression. Primers used for RT-qPCR are listed in Supplementary Table 1.

### Raw data availability

The raw sequence data reported in this paper have been deposited in the Genome Sequence Archive (Chen et al., 2021) in BIG Data Center (Members and Partners, 2022), Beijing Institute of Genomics (BIG), Chinese Academy of Sciences, under accession numbers CRA005953, which are publicly accessible at http://bigd.big.ac.cn/gsa.

## Results

### Transcriptional responses among tissues upon heat treatment

**FIGURE 1:**
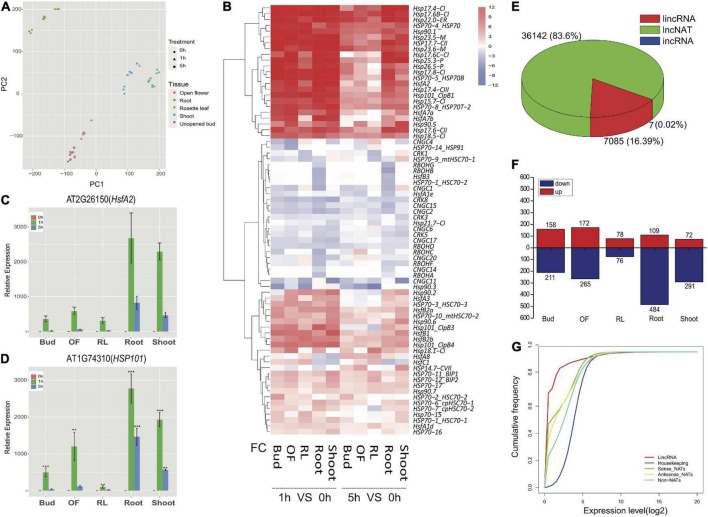
Transcriptomic responses in different tissues and long non-coding RNA expression under heat treatment. **(A)** PCA of transcripts derived from vegetative and reproductive tissues. **(B)** Heatmap for transcripts of identified heat responsive genes. X axis represents the log2 fold change (FC) of transcript abundance between control samples and heat-treated samples. **(C)** qRT-PCR for transcripts of heat response marker gene HSFA2. **(D)** qRT-PCR for transcripts of heat response marker gene HSP101. **(E)** The number of long non-coding RNAs identified under heat treatment. **(F)** The number of differentially expressed lincRNAs in different tissues. **(G)** Cumulative frequency for expressions of lincRNAs, housekeeping genes, sense genes of NATs, antisense gene of NATs and non-NAT genes. *P < 0.05, **P < 0.01, ***P < 0.001 in Dunnett’s two-sided test against 0 h. Error bars indicate SD (*n* = 3).

Strand-specific lncRNA sequencing was used to investigate transcripts of different tissues from young (Shoot and Root) and adult (Rosette leaves, Bud, and Opened flower) plants after 1 and 5 h of heat stress treatment. We obtained around 17G reads per sample, of which 97.7% were successfully mapped (Supplementary Table 2). The correlation coefficient between the three biological replicates was very high (r is around 0.99, Supplementary Figure 1). Principal component analysis (PCA) analysis revealed that the control and treated samples at each time-point were well separated (Figure 1A). Among them, 7.4% (406/5,468) and 4.5% (689/15,094) of the genes were commonly up-regulated and down-regulated between different tissues, respectively (Supplementary Figures 2A,B). Gene Ontology (GO) enrichment analysis showed that genes involved in heat and heat acclimation were enriched for up-regulated gene groups (Supplementary Figure 2C), whereas down-regulated gene groups were enriched with microtubule-based movement, response to karrikin and DNA replication origin binding (Supplementary Figure 2D). Analysis focusing on tissue-specific regulated genes revealed different transcriptomic responses to heat stress among tissues. In young seedlings, transcripts derived from RNA splicing-related genes were specifically enriched in roots (Supplementary Figure 3). By contrast, transcripts involved in tRNA and rRNA processing were induced in shoots after 5-h heat stress (Supplementary Figure 4) indicating differences in post-transcriptional regulation between roots and shoots. In adult plants, GO-terms related to cell cycle, DNA repair, and DNA replication were enriched in down-regulated genes in the reproductive tissues consistent with a previous report (Zhang et al., 2017; Supplementary Figure 5). Figure 1B shows expression patterns of gene families related to signal transduction and tolerance acquisition in heat stress responses. Although the overall patterns were similar among samples, adult plants showed a faster attenuation of induction at the 5-h time point compared to young plants, especially for RL. This observation was confirmed by RT-qPCR in Figures 1C,D. HSFA7b, a positive regulator of heat stress response was not induced in RL, which may contribute to the faster attenuation of heat stress response in this tissue than that of Bud and OF (Figure 1B).

### Identification of heat stress responsive lncRNAs

**FIGURE 2:**
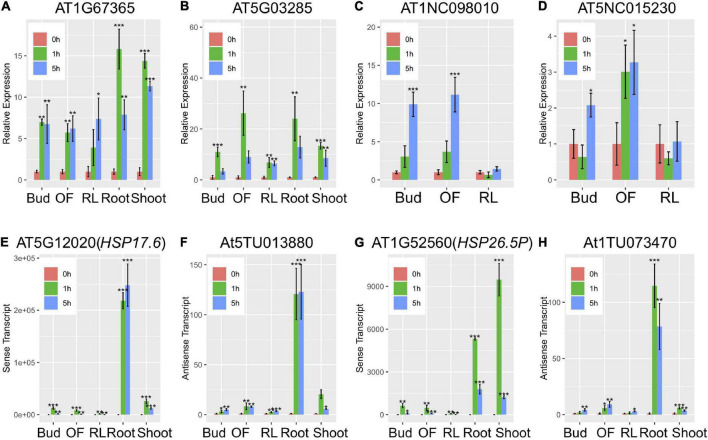
qRT-PCR for lincRNAs and NAT pairs in different WT tissues. LincRNAs including AT1G67365 **(A)**, AT5G03285 **(B)**, AT1NC098010 **(C)**, and AT5NC015230 **(D)**. NAT pairs including HSP17.6CII **(E)**-At5TU013880 **(F)** and HSP25.6 **(G)**-At1TU073470 **(H)**. *P < 0.05, **P < 0.01, ***P < 0.001 in Dunnett’s two-sided test against 0 h. Error bars indicate SD (*n* = 3).

To examine if lncRNAs are involved in heat response, the lncRNA-seq reads were used to assemble new transcripts (Supplementary Table 2). Together with annotated genes and previously released lincRNAs and lncNATs, a total of 43,234 lncRNAs were found to respond to heat treatment with lncNATs being the most abundant (83.6%) (Supplementary Figure 6 and Figure 1E). Comparison of control and treated samples at different time point uncovered 1,189 lincRNAs which were differentially expressed, and heat treatments up-regulated 441 genes encoding these lincRNAs (Fold change, FC ≥ 2; *p* < 0.05) (Figure 1F). Comparing with protein coding genes, the expression levels of lncRNA genes were much lower (Figure 1G). Interestingly, expression levels of most up-regulated lincRNAs were very low in control samples and increased substantially after heat treatment (average 4 times) (Supplementary Figures 7A,B). This result suggested that these lincRNAs might be specifically responsive to heat stress. Venn graph between different tissues showed that the up-regulated lincRNAs are good candidates as tissue-specific regulators (Supplementary Figures 8A,B). RT-qPCR analysis validated the heat-inducibility of 2 lincRNAs that were ubiquitously induced in young and adult plants (Figures 2A,B) and 2 other lincRNAs that were specifically induced in reproductive tissues of adult plants (Figures 2C,D). LincRNAs might regulate its neighboring genes by interacting with chromatin-associated proteins (Chekanova et al., 2007). Because of the limited number of lincRNAs differentially expressed in each sample, the neighboring genes of up-regulated lincRNAs (within 100 kb) were combined for the analysis. GO enrichment analysis suggested that these neighboring genes were enriched in plant defense signaling pathways such as responses to stress and to stimulus (Supplementary Figure 9).

### Expression of natural antisense transcripts pairs during heat treatment

Based on the criterion established in our previous work (Wang et al., 2014), cis-NAT pairs were defined if the overlapping region between sense and antisense transcripts was more than 50 bp. The transcript showing a higher conservation with its rice counterpart would be designated as the sense transcript and the opposite strand would be the antisense transcript. If both strands did show any sequence conservation with rice transcripts, the transcripts expressed in most samples will be regarded as the sense transcripts. Combined with our previous work (Wang et al., 2014), the final cis NAT pairs contained 36,317 pairs (mRNA-mRNA, 1,247; mRNA-lncRNA, 32,813; lncRNA-lncRNA, 2,257), comprising 22,876 annotated mRNAs and 37,165 novel lncRNA transcripts (Supplementary Table 3). According to the transcription direction, NAT pairs could be further classified into three different types: enclosed, convergent, or divergent (Wang et al., 2014). In our study, 64.5% (23,420 out of 36,317) of NAT pairs were enclosed (Supplementary Table 3), which is similar with maize (Xu et al., 2017) and rice (Lu et al., 2012); by contrast, most NAT pairs in *Brassica rapa* (Yu et al., 2013) are convergent.

**FIGURE 3:**
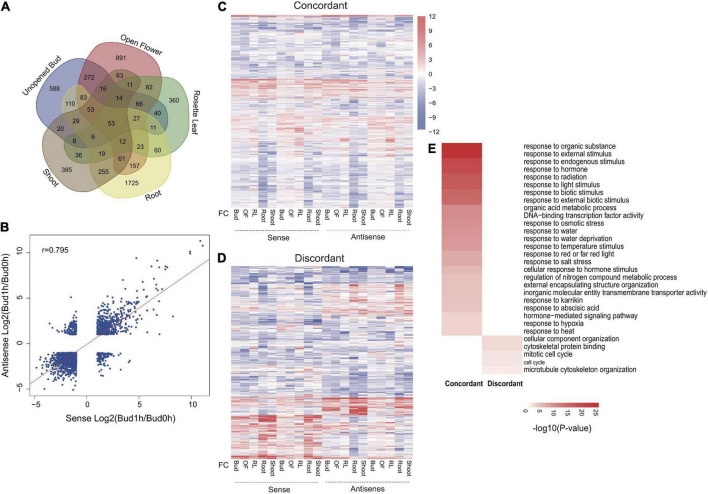
Response of NAT pairs under heat treatment. **(A)** Venn graph for differentially expressed genes of NATs between different tissues. **(B)** Correlation analysis using expression level of sense and antisense genes of NAT pairs under heat treatment. **(C)** Heatmap for concordant NATs pairs with response to heat stress. **(D)** Heatmap for discordant NATs pairs with response to heat stress. **(E)** Top 30 specifically enriched GO terms for concordant and discordant NAT pairs.

Furthermore, we identified heat-responsive NAT pairs using the following criteria: (1) a 2-fold change comparing expression level between control and treated samples at any tissues for both sense and antisense transcripts; and (2) a *P* < 0.05. A total of 5,536 NATs were identified as heat-responsive transcripts. Interestingly, heat-responsive NATs were expressed from many HSP and HSF genes in different tissues and 2 NATs were validated by RT-qPCR (Figures 2E–H and Supplementary Table 4). However, many other heat-regulated transcripts showed a clear tissue-specific preference [71.33%; 3949 (588 + 385 + 1,725 + 360 + 891) out of 5,536] (Figure 3A). Comparing among different tissues, most heat-responsive NAT pairs were preferentially expressed in roots (1,725) (Figure 3A). According to the expression correlation between sense and antisense transcripts, we further divided these heat-regulated NAT pairs into two clusters: concordant (positive correlation) and discordant (negative correlation). By dynamic transcriptome comparisons, fold change of sense and antisense expression was significantly correlated (Figure 3B). Upon heat stress, increased expression of most of the up-regulated genes was accompanied by elevated antisense transcription as well (Figure 3B). We identified 4,480 concordant NAT pairs and 1,056 discordant NAT pairs potentially involved in heat signaling pathways and responses (Figures 3C,D). In order to explore functional differences between concordant and discordant pairs, GO enrichment analysis was performed for genes encoding these NAT pairs. Interestingly, genes coding for hormone (GO: 0009725-response to hormone), transcription factors (GO: 0003700-DNA-binding transcription factor activity) and heat (GO: 0009408-response to heat) were only over-represented in concordant pairs whereas genes encoding for cell cycle (GO: 0000278-mitotic cell cycle; GO: 0007049-cell cycle) were only over-represented in discordant pairs (Figure 3E).

### Transcriptional regulation of genes for heat-inducible lincRNAs and natural antisense transcripts

**FIGURE 4:**
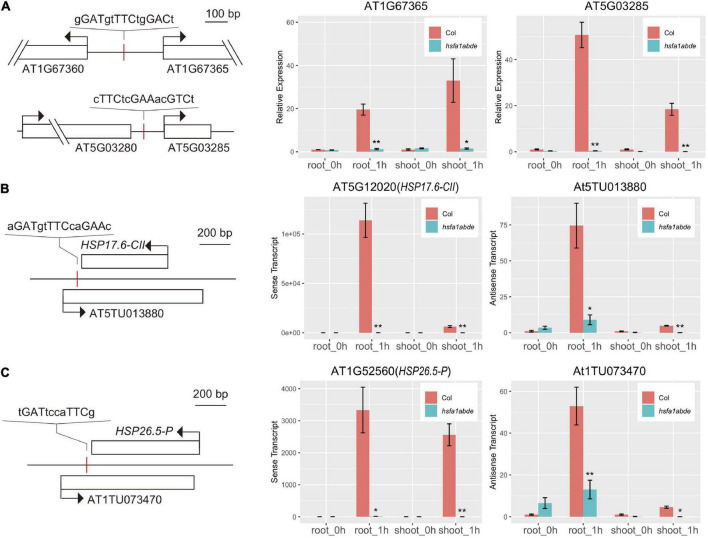
Position of *HSFA1*-binding sites and qRT-PCR for several lincRNAs and NAT pairs in *hsfa1abde* mutant. **(A)** Schematic diagram of structure and qRT-PCR results of genes encoding heat-inducible lincRNAs (AT1G67365 and AT5G03285). **(B)** Schematic diagram of structure and qRT-PCR results of genes encoding heat-inducible NAT pairs (HSP17.6CII-At5TU013880). **(C)** Schematic diagram of structure and qRT-PCR results of genes encoding heat-inducible NAT pairs (HSP25.6-At1TU073470). Red lines and open boxes indicate positions of HSE found around ChIP-seq peaks and gene bodies, respectively. Sequence of each HSE was shown above the red lines. At1TU073470 has non-canonical HSE (nGAAnnnnTTCn), whereas other genes have canonical HSE (double or triple inverted repeats of nGAAn). One conserved adenine in nGAAn and one conserved thymine in nTTCn are exchangeable with other nucleotides. *P < 0.05, **P < 0.01 in two-tailed Student’s *t*-tests between Col and *hsfa1abde*. Error bars indicate SD (*n* = 3).

In contrast to protein-coding genes, transcriptional regulation for lincRNA and NAT is not well understood. In response to heat stress, *HSFA1* functions as a dominant regulator of transcriptional activation, especially, during early stages of the response (within 1 h) (Yoshida et al., 2011). To examine whether *HSFA1* was involved in the induction of lncRNA, we analyzed a public ChIP-seq data (Albihlal et al., 2018) of *HSFA1b* and found that genes encoding heat-inducible lincRNAs in Figures 2A,B showed peaks of ChIP signal with HSF-binding sites (Heat shock element, HSE) in their promoter regions (Figure 4A). Furthermore, about half of the HSP and HSF genes listed in Supplementary Table 4 also had ChIP peaks associated with promoters or 5′ regions of their NAT genes. Canonical and non-canonical HSEs (Nieto et al., 2018) were found at the ChIP-peak regions around NAT genes in Figures 2, 4B,C, suggesting that expression of lincRNA and NAT was induced by *HSFA1* upon heat shock. Consistent with this hypothesis, RT-qPCR analysis showed that 4 representative lncRNAs were not induced in the *hsfa1* quadruple mutant (*hsfa1abde*) after 1 h heat stress (Figures 4A–C).

### Histone modifications on genes for natural antisense transcript pairs

**FIGURE 5:**
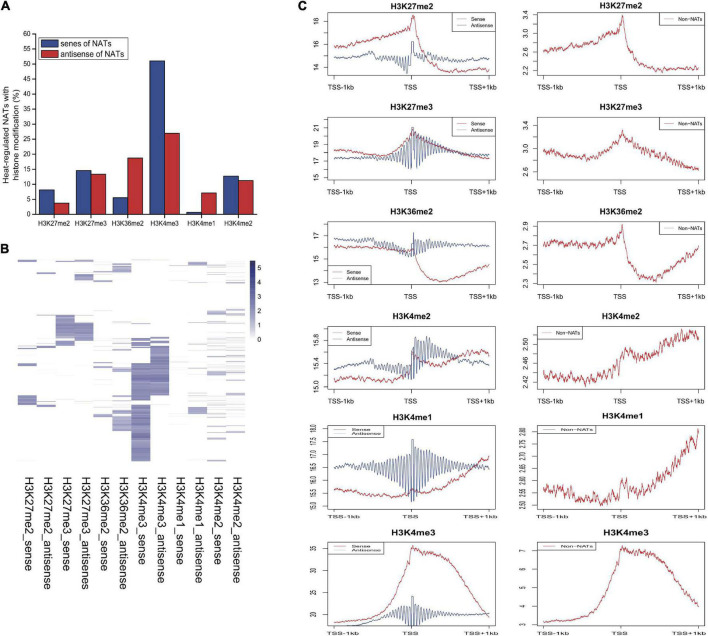
Histone modifications associated with NAT pairs. **(A)** Associated of six different histone modifications for heat-regulated NAT genes. **(B)** Heatmap of histone modification levels for genes of NATs. **(C)** The degree of six different histone markers around transcription start sites (TSS) of NAT pairs.

The identification of numerous heat-responsive NATs led us to further focus on epigenetic regulation mechanism of genes encoding these transcripts. To investigate whether histone modification was associated with genes for these heat responsive NAT pairs, 6 different kinds of histone modifications from public ChIP-chip data, including H3K27me2, H3K27me3, H3K36me2, H3K4me1, H3K4me2, and H3K4me3, were used to identify potential enriched histone marks by TileMap (Oh et al., 2008; Zhang et al., 2009; Ji et al., 2011). Histone mark peaks were aligned around transcription start sites (TSS) (±1000-nt region) of genes for sense and antisense of heat responsive NAT pairs. We found that many heat-regulated genes encoding NATs (∼50% for sense and ∼30% for antisense) were enriched with histone markers (Figure 5A and Supplementary Table 5). Genes for both sense and antisense NATs had a slight preference for H3K4me3 (Figure 5A). To explore if heat stress may also regulate the expression of NATs by histone modifications, we attempted to check whether histone levels were correlated with transcription changes of genes for these heat responsive NATs. From the heatmap for histone levels of NAT genes, we found that, compared to other histone markers, H3K27me3 and H3K4me3 was more significantly associated with genes for both sense and antisense of heat responsive NATs (Figure 5B). Furthermore, similar to protein-coding genes, H3K36me2 was significantly enriched in the promoter region near the transcription start site (Figure 5C). We noted that NAT genes were more enriched in H3K4me2 and H3K4me1 around 1kb flanking sequences downstream of the TSS (Figure 5C).

### Heat responsive genes for natural antisense transcript pairs were associated with deoxyribonucleic acid methylation

**FIGURE 6:**
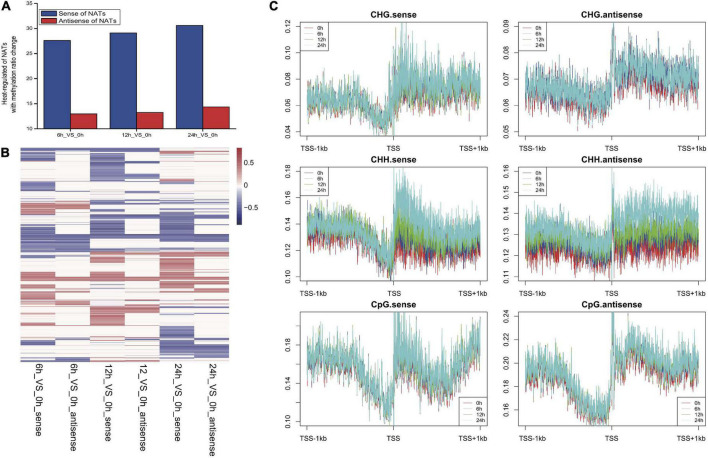
Methylation associated with NAT genes. **(A)** Degree of methylation associated with heat-regulated NAT genes. **(B)** Heatmap of percentage of methylation of NAT genes at different time points. **(C)** The degree of different methylation types around transcription start sites (TSS) of NAT pairs.

Previous genome-wide profiling of methylation showed a gradual reduction in the level of methylation during heat stress treatment, especially at the end of heat stress and returning to control conditions (Korotko et al., 2021). However, whether methylation was associated with non-coding genes derived from the antisense strand of coding genes has not been explored. In order to explore the change of methylation of genes for heat responsive NAT pairs, public ChIP-seq data for control and heat treatment samples were studied (Korotko et al., 2021), which was performed on leaves of wild-type *A. thaliana* seedlings exposed to 0, 6, 12, and 24 h of high temperature. We found that many heat-regulated NAT genes (∼30% for sense and ∼15% for antisense) were associated with methylation under heat treatment (Figure 6A). Meanwhile, genes for sense and antisense of NATs have similar methylation pattern at different time points during heat stress treatment (Figure 6B). Moreover, more NAT genes have decreased DNA methylation level under heat stress (42%-up vs. 58%-down at 6 h, 40%-up vs. 60%-down at 12 h and 38%-up vs. 62%-down at 24 h for sense of NATs) (Supplementary Table 6). Among NAT genes, the most methylated cytosines were found in the CpG context, followed by CHH and then CHG (Figure 6C). Interestingly, CHH, CHG and CpG were relatively hypermethylated around the transcription start site (Figure 6C).

### Enrichment of small ribonucleic acids in complementary sequences of natural antisense transcripts

**FIGURE 7:**
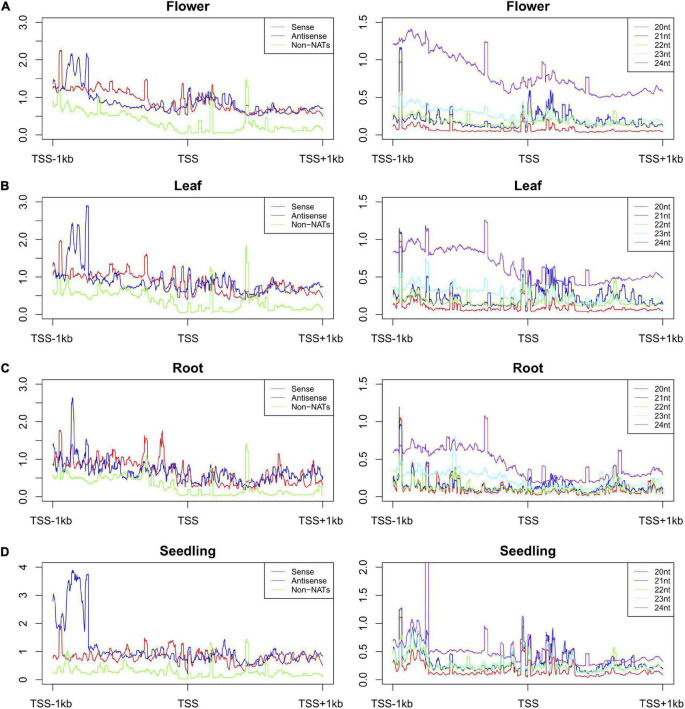
Distribution of small RNAs in flower **(A)**, leaf **(B)**, root **(C)**, and seedling **(D)** around transcription start sites (±1000-bp) of sense and antisense genes of NATs and non-NAT genes.

Previous studies have demonstrated that NAT pairs might be associated with or regulated by small RNAs (smRNAs) (Borsani et al., 2005; Wang et al., 2014). In this study, we used our previously published smRNA sequencing data, including seedlings, leaves, flowers, and roots to investigate whether smRNAs were associated with NAT pairs (Wang et al., 2011). Figure 7 depicts smRNA enrichment around transcription start sites (±1000-nt region). The number of smRNA reads was 1.52 for sense, 1.59 for antisense and 0.63 for non-NATs (Supplementary Table 7). Moreover, smRNAs were significantly enriched in the promoter regions for NAT genes (Figure 7). In addition, 24-nt smRNA were more abundant compared with other smRNAs (Figure 7). Similar results were found for 2000-bp promoter region (Supplementary Figure 10). Potential nat-siRNAs were defined as all 20- to 24-nt smRNAs which could be totally mapped to the complementary regions of NAT pairs. In total, around 15,000 NAT pairs may act as precursors of nat-siRNAs (Supplementary Table 8). Among them, around 3,000 NATs pairs were responsive under heat treatment. Our results uncover a possible novel role for NAT pairs in nat-siRNA-mediated heat response.

## Discussion

Although genome-wide identification of lncRNA has been conducted using various strategies (Jin et al., 2021), the function of most of them remain unclear, especially those expressed under environmental stress. In this study, we attempted to identify NATs in *Arabidopsis* and explore their potential function under heat treatment. Using strand-specific lncRNA-seq and our previous results (Liu et al., 2012; Wang et al., 2014), we identified 36,317 NAT pairs, containing 22,876 annotated mRNAs and 37,165 novel lncRNA transcripts.

### Natural antisense transcripts are regulated in a spatial-temporal manner under heat treatment

Our transcriptome analysis revealed that many heat-responsive coding and non-coding genes were differentially regulated among tissues and at different time points following heat treatment. Previous works reported that expression of lincRNA and NAT is induced in a highly tissue- and stress-specific manner (Jin et al., 2013, 2021). This observation also holds true for heat stress conditions indicating potential roles of non-coding transcripts in adaptive growth at elevated temperatures. Considering that high temperature has differential impact on different organs, it is not surprising to see organ-specific adaptive responses at the gene expression level. For example, being the most vulnerable to heat stress, flowers are protected by the tissues-specific activation of unfolded protein response pathway under heat stress conditions (Fragkostefanakis et al., 2016; Zhang et al., 2017). Although the function remains to be elucidated, we have identified specific heat-responsive lincRNAs that are induced in reproductive tissues (Figures 2C,D). Furthermore, reproductive tissues (Bud and OF) expressed more NATs than vegetative tissue (RL) (Figure 3A). These observations suggest the presence of a flower-specific protective mechanism operating through non-coding transcripts. We also found that heat-responsive NATs were preferentially expressed in roots (Figure 3A). Compared with aerial parts, how root copes with heat stress is not well understood. However, root-specific mechanism could be expected because soil may function as a buffer of temperature change resulting in a milder and slower elevation of root temperature compared to that experienced by shoots under natural situations (D E Lima et al., 2021). The general function of an antisense transcript is considered to be negative regulation of its sense transcript. Therefore, root cells might use NAT as a brake to attenuate unrestrained response reflecting the underground heat conditions.

Although further investigation is required about tissue-specific regulation of lncRNA expression, our analysis showed HSFA1 as a potential regulator of heat-dependent induction of lncRNAs. Previous work reported that HSFA1 induces NAT of *HSFB2a* during heat stress, which contributes to the negative regulation of *HSFB2a* expression (Wunderlich et al., 2014). Analyses of public ChIP-seq data and gene expression in *hsfa1* quadruple mutant indicates that HSFA1 also induces lincRNAs and NATs of HSP genes (Figures 4A–C). As represented by the enrichment of heat response-related genes and transcription factor genes among concordant NAT pairs, many HSP and HSF genes possess heat-responsive NATs (Supplementary Table 4). Whereas HSFA1 induces sense transcripts of HSP and HSF, this transcription factor may also fine-tune their expression through the simultaneous induction of their cognate antisense transcripts.

### Chromatin marks of heat-responsive natural antisense transcript genes

Epigenetic marks including histone modification and methylation play important roles in chromatin structure and gene function (Charron et al., 2009; Kharchenko et al., 2011; Korotko et al., 2021). Among them, H3K4me3 and H3K36me3 are known as active histone marks, and H3K4me3- and H3K36me3-marked genes are known to have relatively higher expression levels (Li et al., 2007). By contrast, H3K27me3 is primarily enriched on promoters and gene bodies of repressed genes. Mutual antagonism between H3K27me3 and DNA methylation suggests a dynamic crosstalk between these epigenetic marks that could help maintain appropriate gene expression levels (Jermann et al., 2014; Meehan and Pennings, 2017). Several studies have revealed that some antisense transcripts of NATs may regulate histone modification or methylation level at specific genomic loci (Wang et al., 2014; Xu et al., 2017). Overall, in our study, the relatively low levels of H3K27me2/3 of NATs in Arabidopsis are consistent with active chromatin for heat-response whereas these heat-responsive NAT genes were associated with relatively high H3K4me3 levels. Moreover, more NAT genes have decreased DNA methylation level under heat stress (42%-up vs. 58%-down at 6 h, 40%-up vs. 60%-down at 12 h and 38%-up vs. 62%-down at 24 h for sense of NATs) (Supplementary Table 6). Taken together, our results indicate that genes for heat responsive NAT and host transcripts are associated with positive histone marks, which is consistent with the dynamic expression change upon heat stress. These results provide a global view of the complex relationship between expression of NATs and chromatin modifications of their encoding genes.

Meanwhile, H3K4me3 levels of genes for sense transcripts of NATs were much higher than those for genes encoding antisense transcripts. Our result may provide support on possible antisense-mediated histone modification or changes in methylation level of genes for sense transcripts during heat treatment. However, whether the changes in chromatin marks are triggered by heat treatment alone or indirectly mediated by the action of its antisense transcripts is still unknown.

### Possible regulation mechanism of natural antisense transcript pairs in response to heat

Previous studies revealed a complicated relationship between sense and antisense transcripts in both yeast and humans (Xu et al., 2011; Balbin et al., 2015). One accepted hypothesis was double stranded RNA could be generated from the sequence overlapping region of sense and antisense transcripts. Borsani et al. have found that the SRO5-P5CDH nat-siRNAs together with the proteins encoded by *P5CDH* and *SRO5* are key components of a regulatory loop controlling ROS production and stress response (Borsani et al., 2005). In Arabidopsis, there is a sixfold enrichment of cis-NATs in the sequence overlapping regions compared with non-overlapping regions (Zhang et al., 2013). In maize, smRNAs are also found to be enriched in NAT pairs, especially in the overlapping regions (Xu et al., 2017). Similarly, in our study, smRNAs are also significantly enriched in overlapping regions of NAT pairs (Supplementary Table 7). Moreover, around 3,000 heat-responsive NAT pairs may act as potential precursors of nat-siRNAs (Supplementary Table 8). These results reveal a possible novel role for NAT pairs in nat-siRNA-mediated heat response. Whereas the mechanism of miRNA-mediated thermotolerance and heat stress memory has been extensively analyzed (Guan et al., 2013; Stief et al., 2014; Lin et al., 2018), how nat-siRNA is involved in heat stress response is not well-understood. Nevertheless, the thermosensitive phenotype of mutants related to small RNA biogenesis pathway is consistent with the importance of nat-siRNA in high temperature adaptation (Popova et al., 2013). Future analysis focusing on global identification of heat-responsive smRNA will provide further insight into the role of nat-siRNA in heat stress response.

## Data availability statement

The datasets presented in this study can be found in online repositories. The names of the repository/repositories and accession number(s) can be found below: http://bigd.big.ac.cn/gsa, CRA005953

## Author contributions

JJ, NO, H-WW, and N-HC designed the experiments. NO and H-WW executed the molecular experiments. JJ and XH performed the bioinformatic analysis. JJ, NO, and N-HC wrote the manuscript. All authors contributed to the article and approved the submitted version.

## Supplementary material

Supplementary Figure 1 Correlation between different biological replicates for each sample.

Supplementary Figure 2 Enrichment results for differentially expressed genes under heat treatment. **(A)** Venn graph for up-regulated genes in different samples. **(B)** Enriched GO terms for commonly up-regulated genes between reproductive tissues and vegetative tissues. **(C)** Venn graph for down-regulated genes in different samples. **(D)** Enriched GO terms for commonly down-regulated genes between reproductive tissues and vegetative tissues.

Supplementary Figure 3 GO Enrichment results for specific up-regulated genes in root tissues under heat treatment. **(A)** Enriched GO terms for root specific up-regulated genes under heat treatment at 1 h. **(B)** Enriched GO terms for root specific up-regulated genes under heat treatment at 5 h.

Supplementary Figure 4 GO Enrichment results for specific up-regulated genes in shoot tissues under heat treatment. **(A)** Enriched GO terms for shoot specific up-regulated genes under heat treatment at 1 h. **(B)** Enriched GO terms for shoot specific up-regulated genes under heat treatment at 5 h.

Supplementary Figure 5 GO Enrichment results for down-regulated genes between reproductive tissues (Bud and OF) and vegetative tissues (RL). **(A)** Venn graph for down-regulated genes between reproductive tissues (Bud and OF) and vegetative tissues (RL). **(B)** Enriched GO terms for commonly down-regulated genes between reproductive tissues (Bud and OF) and vegetative tissues (RL). **(C)** Enriched GO terms for specific down-regulated genes in reproductive tissues (Bud and OF). **(D)** Enriched GO terms for specific down-regulated genes in vegetative tissues (RL).

Supplementary Figure 6 Pipeline for lncRNA identification in Arabidopsis.

Supplementary Figure 7 Heatmap and boxplot for up-regulated lincRNAs under heat treatment. **(A)** Heatmap for up-regulated lincRNAs under heat treatment. X axis represents the log2 fold change (FC) in transcript abundance between control sample and heat treated sample. **(B)** Boxplot for up-regulated lincRNAs under heat treatment.

Supplementary Figure 8 Venn graph for differentially expressed lincRNAs and lncNATs under heat treatment. **(A)** Venn graph for up-regulated lincRNAs under heat treatment. **(B)** Venn graph for down-regulated lincRNAs under heat treatment. **(C)** Venn graph for up-regulated lncNATs under heat treatment. **(D)** Venn graph for down-regulated lncNATs under heat treatment.

Supplementary Figure 9 Enrichment GO terms for neighboring genes of differentially expressed genes encoding lincRNAs under heat treatment.

Supplementary Figure 10 Distribution of small RNAs in flower **(A)**, leaf **(B)**, root **(C)** and seedling **(D)** around transcription start sites (± 2000-bp) of sense and antisense genes of NATs and non-NAT genes.

## Abbreviations

lncRNA: long non-coding RNA
lincRNA: long intergenic non-coding RNA
smRNA: Small RNA
NAT: Natural antisense transcript
incRNA: intronic RNA
HSF: Heat shock transcription factor
HSPs: heat shock proteins
COOLAIR: Cold-assisted intronic non-coding RNA
FLC: FLOWERING LOCUS C
CLF: CURLY LEAF
AG: AGAMOUS.
