# Supplementary material for: Tissue-specific transcriptomic analysis uncovers potential roles of natural antisense transcripts in Arabidopsis heat stress response
Source: Front Plant Sci. 2022 Sep 8;13:997967. doi: 10.3389/fpls.2022.997967 (PMC9498583; doi:10.3389/fpls.2022.997967)
Supplement: Supplementary Figure 1 — Correlation between different biological replicates for each sample. [file Image_1.PDF]

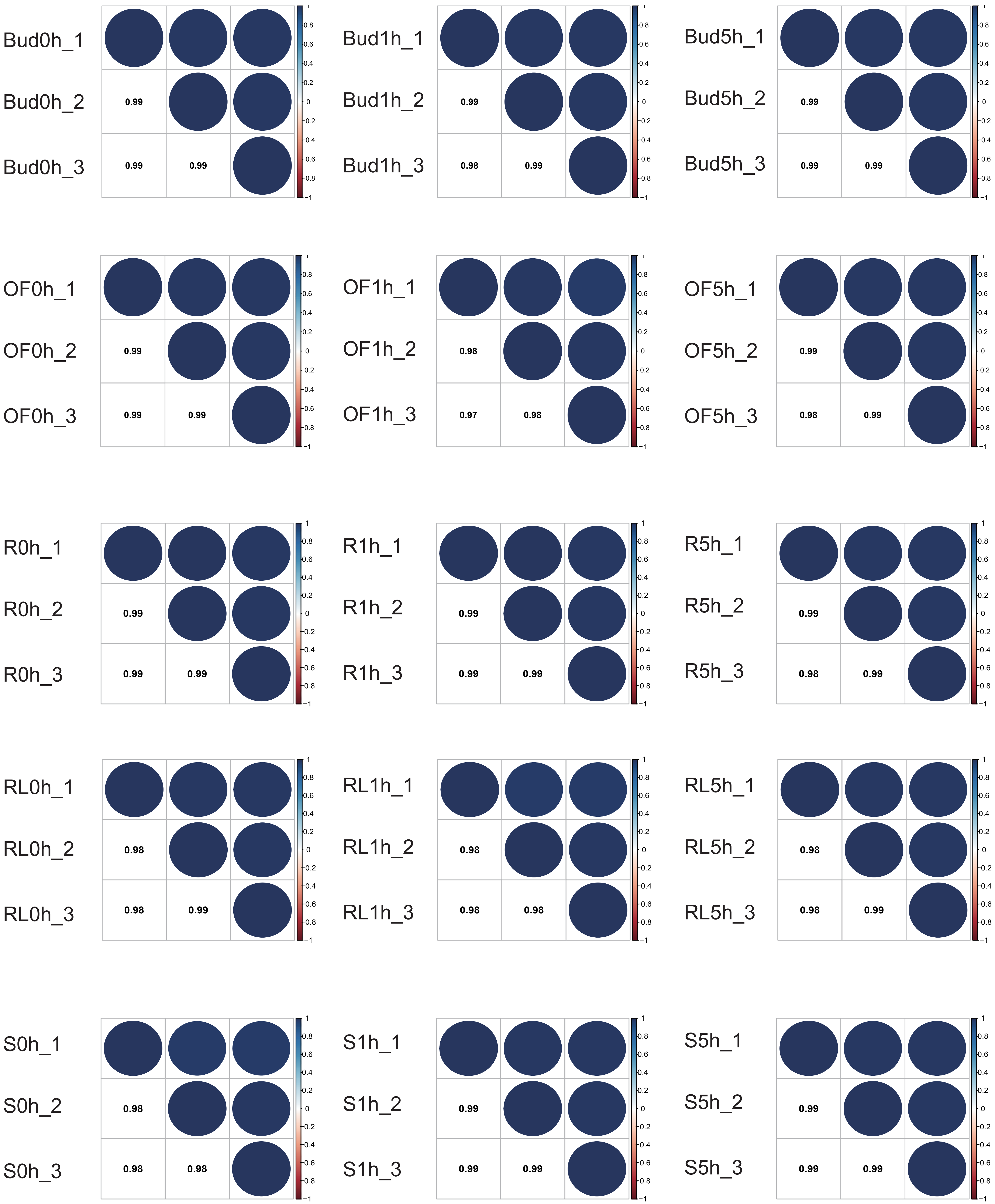

Bud0h_1
Bud0h_2
Bud0h_3
0.99
0.99
0.99
Bud1h_1
Bud1h_2
Bud1h_3
0.99
0.98
0.99
Bud5h_1
Bud5h_2
Bud5h_3
0.99
0.99
0.99
OF0h_1
OF0h_2
OF0h_3
0.99
0.99
0.99
OF1h_1
OF1h_2
OF1h_3
0.98
0.97
0.98
OF5h_1
OF5h_2
OF5h_3
0.99
0.98
0.99
R0h_1
R0h_2
R0h_3
0.99
0.99
0.99
R1h_1
R1h_2
R1h_3
0.99
0.99
0.99
R5h_1
R5h_2
R5h_3
0.99
0.98
0.99
RL0h_1
RL0h_2
RL0h_3
0.98
0.98
0.99
RL1h_1
RL1h_2
RL1h_3
0.98
0.98
0.98
RL5h_1
RL5h_2
RL5h_3
0.98
0.98
0.99
S0h_1
S0h_2
S0h_3
0.98
0.98
0.98
S1h_1
S1h_2
S1h_3
0.99
0.99
0.99
S5h_1
S5h_2
S5h_3
0.99
0.99
0.99
