# Supplementary figures and images for: Tissue-specific transcriptomic analysis uncovers potential roles of natural antisense transcripts in Arabidopsis heat stress response
Source: Front Plant Sci. 2022 Sep 8;13:997967. doi: 10.3389/fpls.2022.997967 (PMC9498583; doi:10.3389/fpls.2022.997967)

A

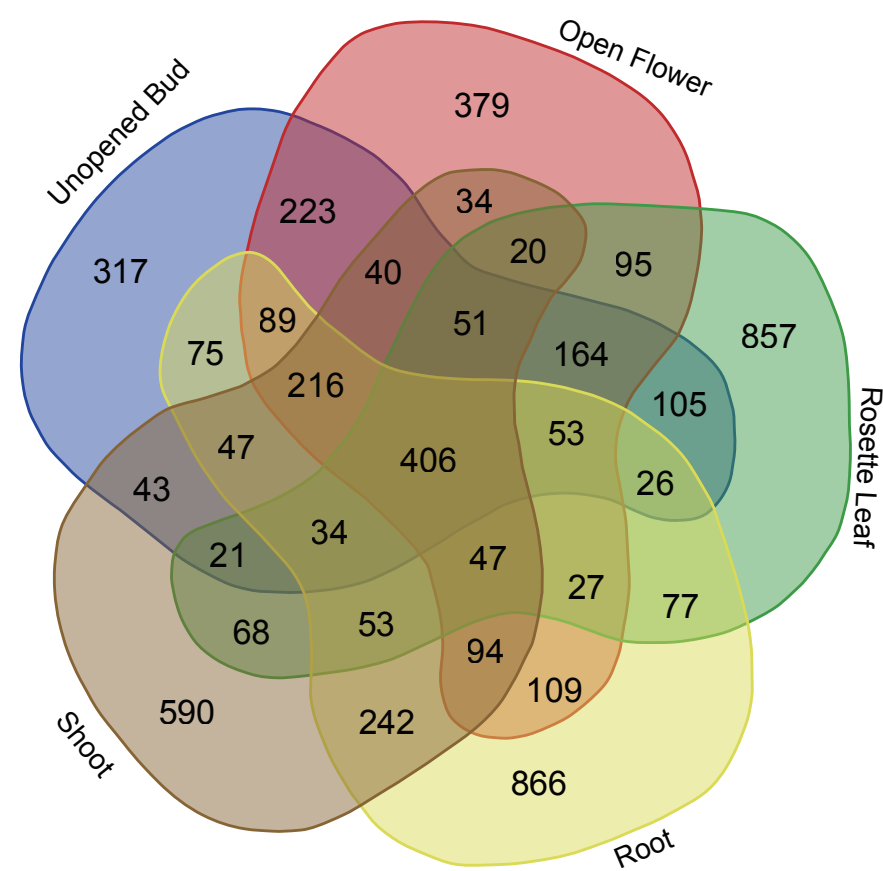

B

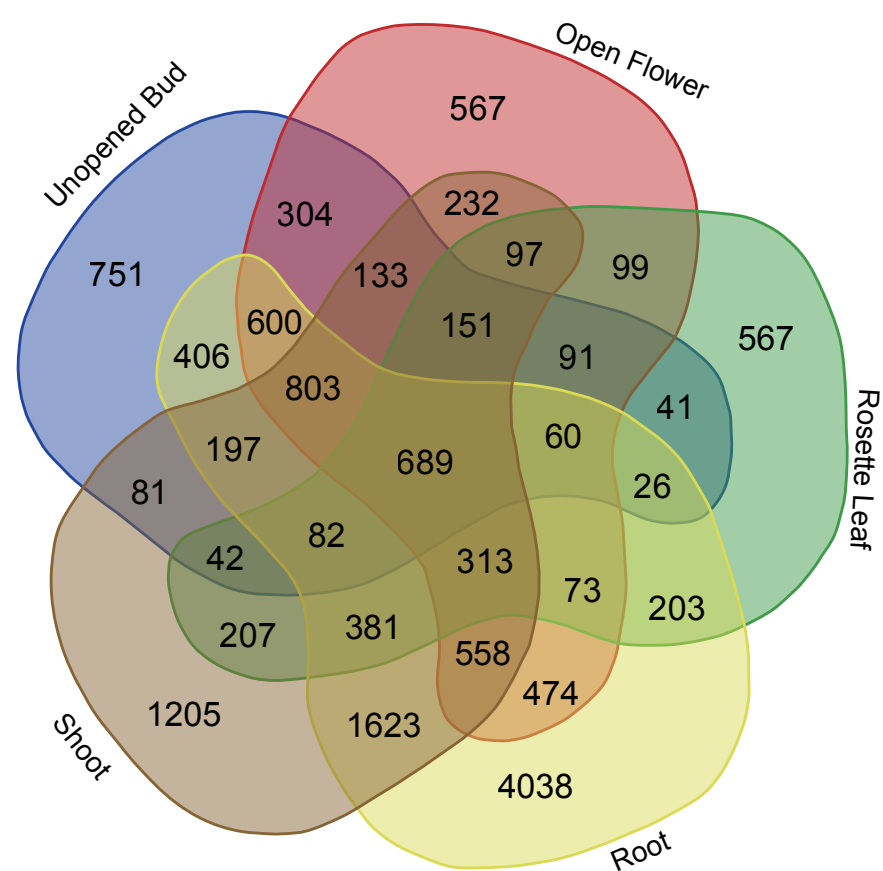

C

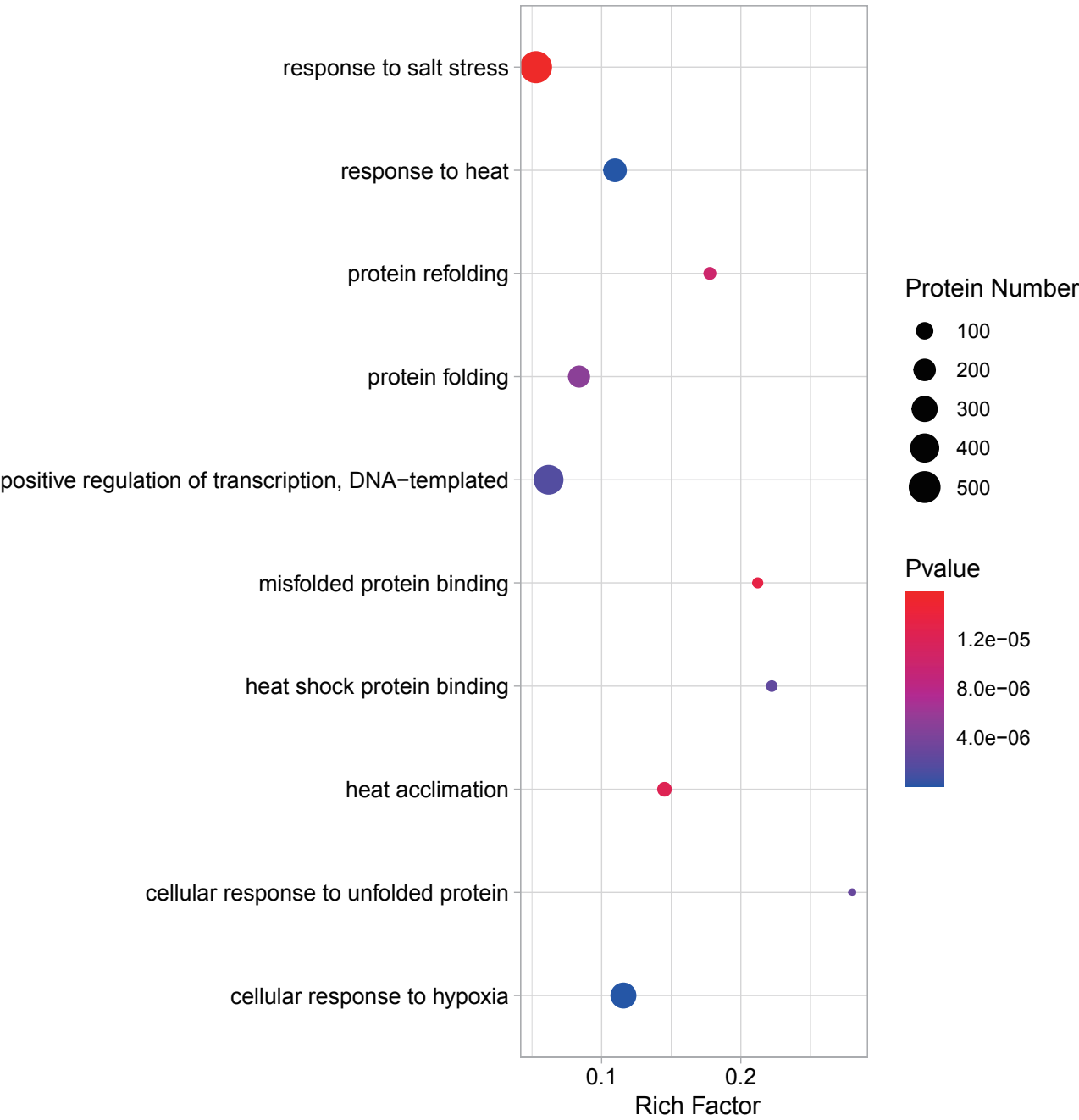

D

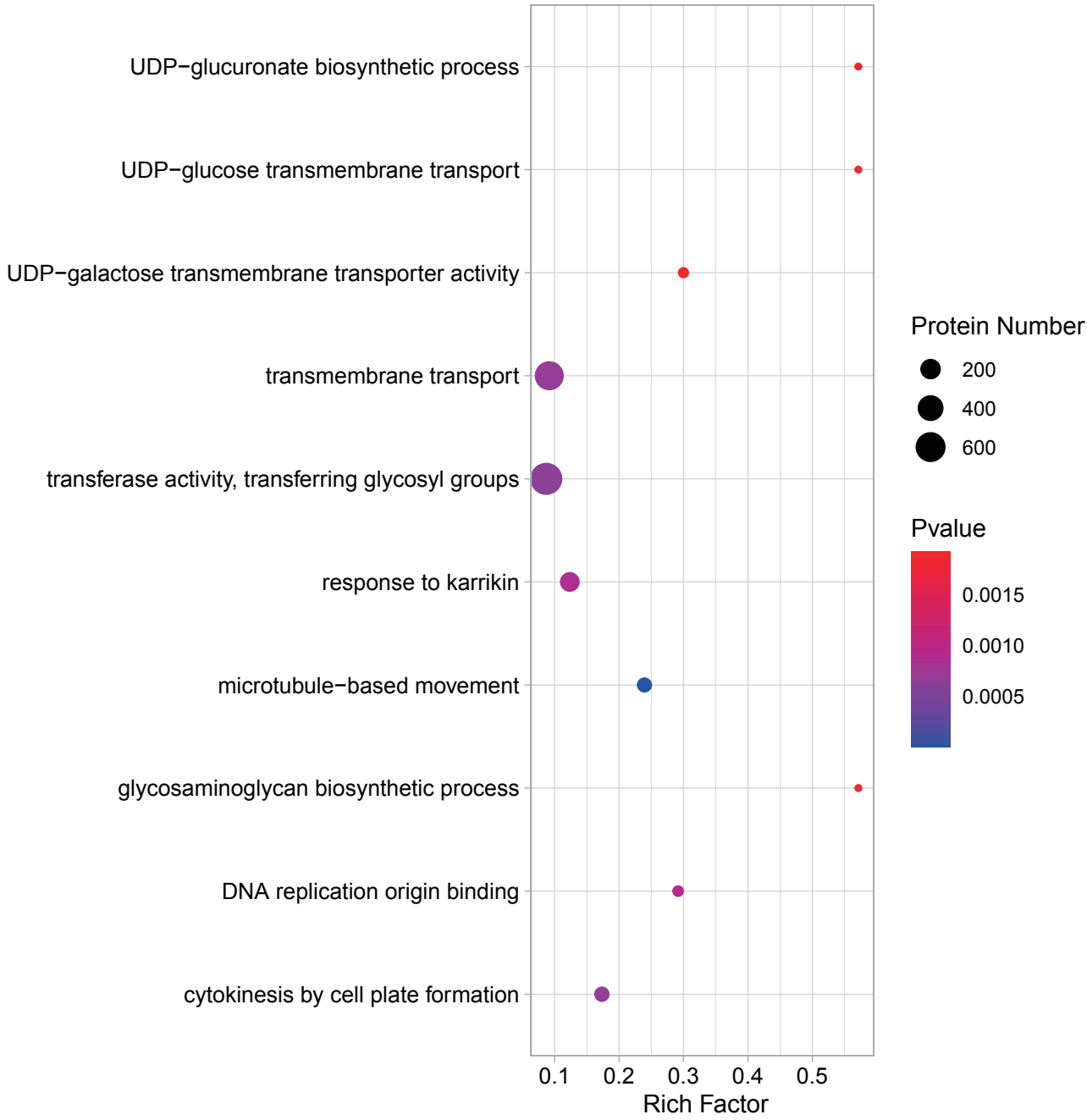

Supplement: Supplementary Figure 2 — Enrichment results for differentially expressed genes under heat treatment. (A) Venn graph for up-regulated genes in different samples. (B) Enriched GO terms for commonly up-regulated genes between reproductive tissues and vegetative tissues. (C) Venn graph for down-regulated genes in different samples. (D) Enriched GO terms for commonly down-regulated genes between reproductive tissues and vegetative tissues. [file Image_2.PDF]

A

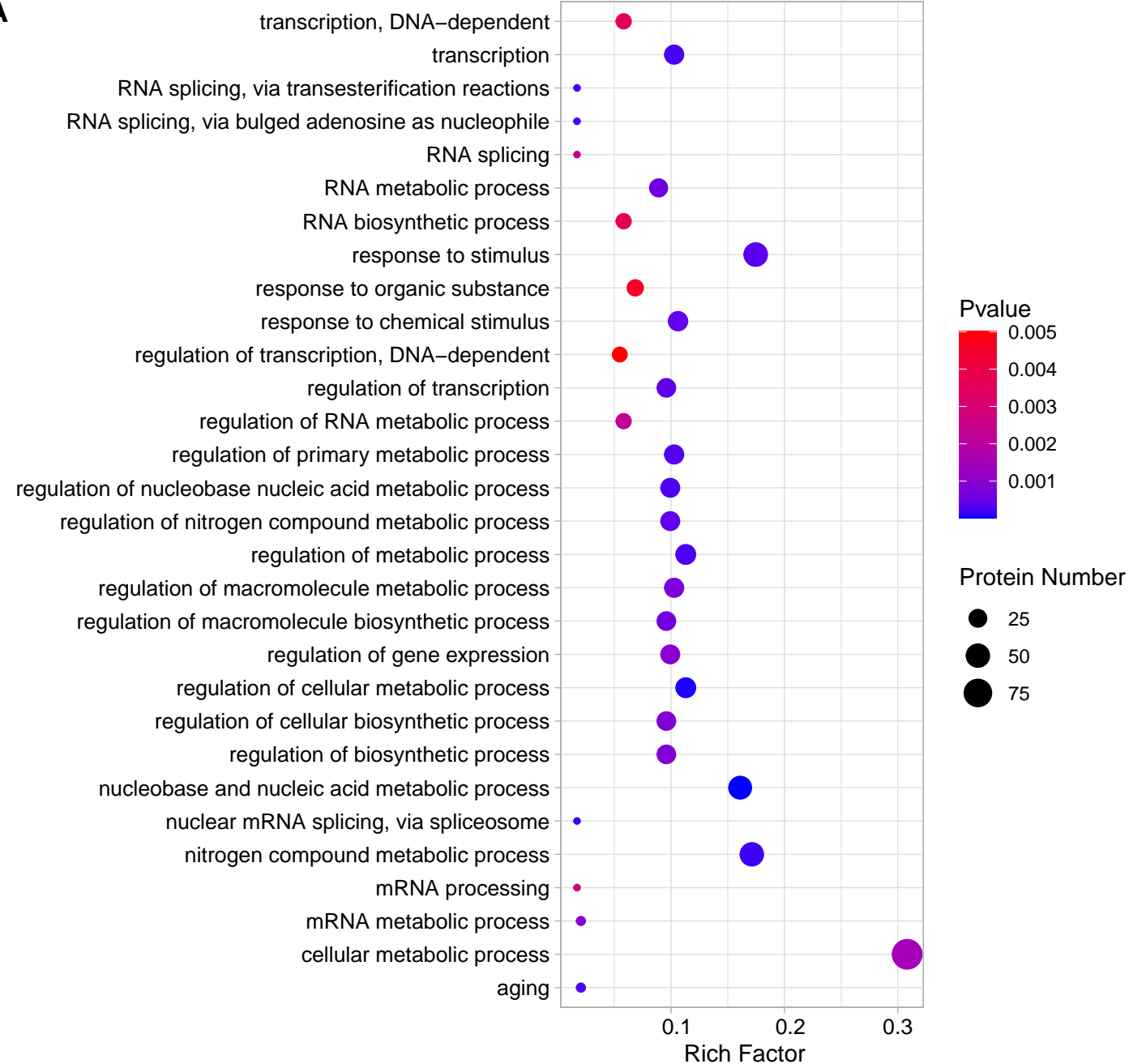

B

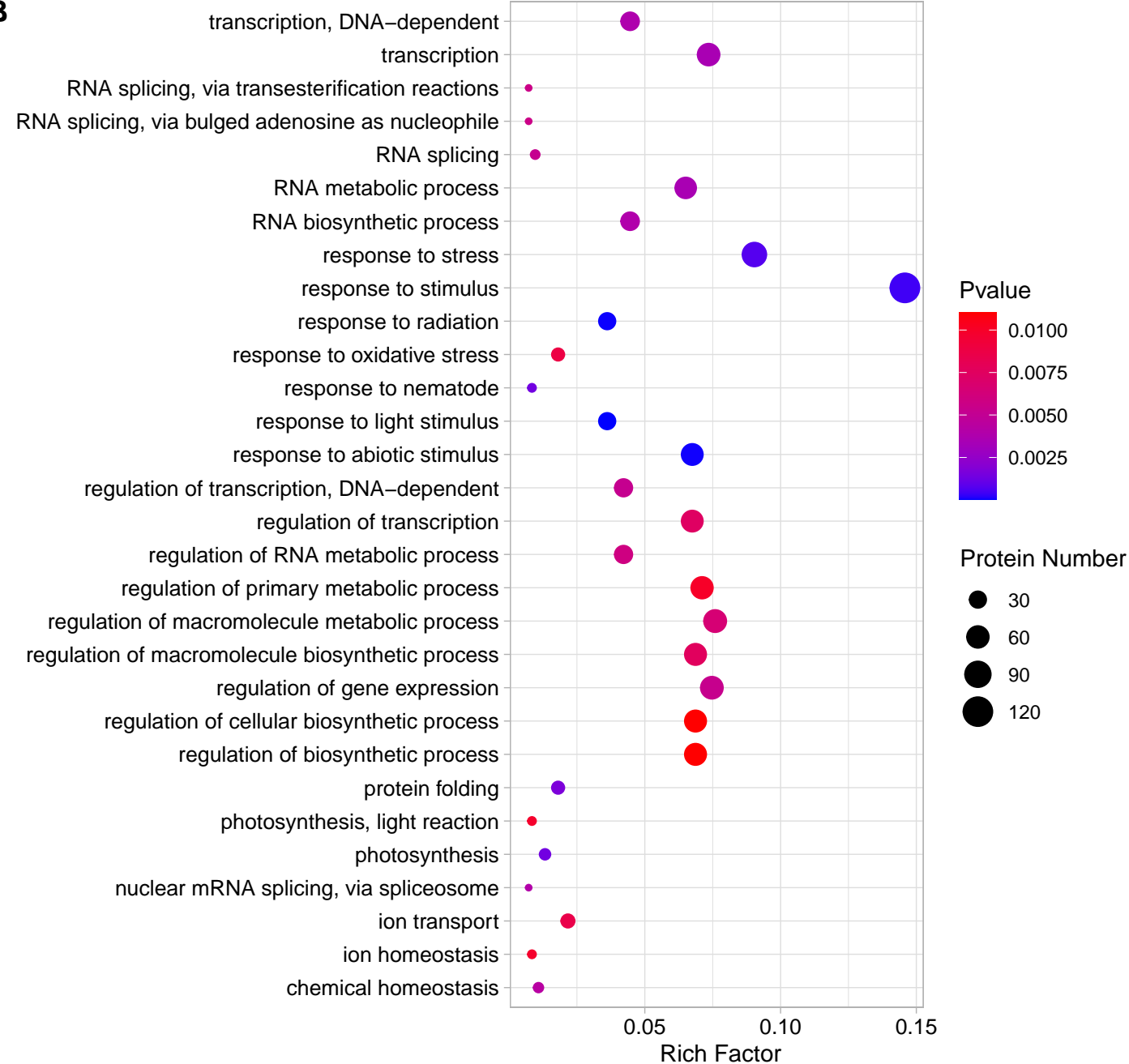

Supplement: Supplementary Figure 3 — GO Enrichment results for specific up-regulated genes in root tissues under heat treatment. (A) Enriched GO terms for root specific up-regulated genes under heat treatment at 1 h. (B) Enriched GO terms for root specific up-regulated genes under heat treatment at 5 h. [file Image_3.PDF]

**A**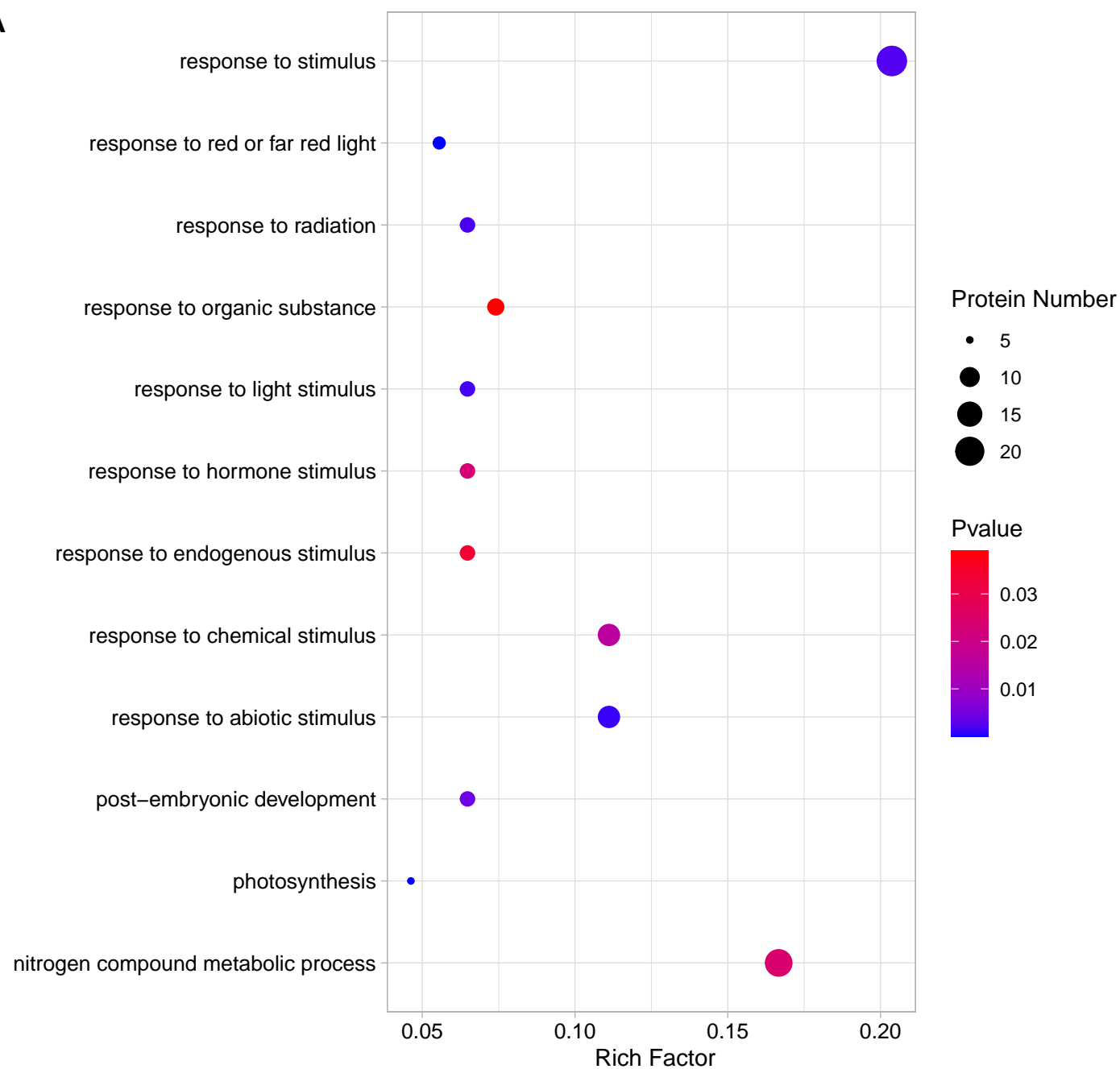**B**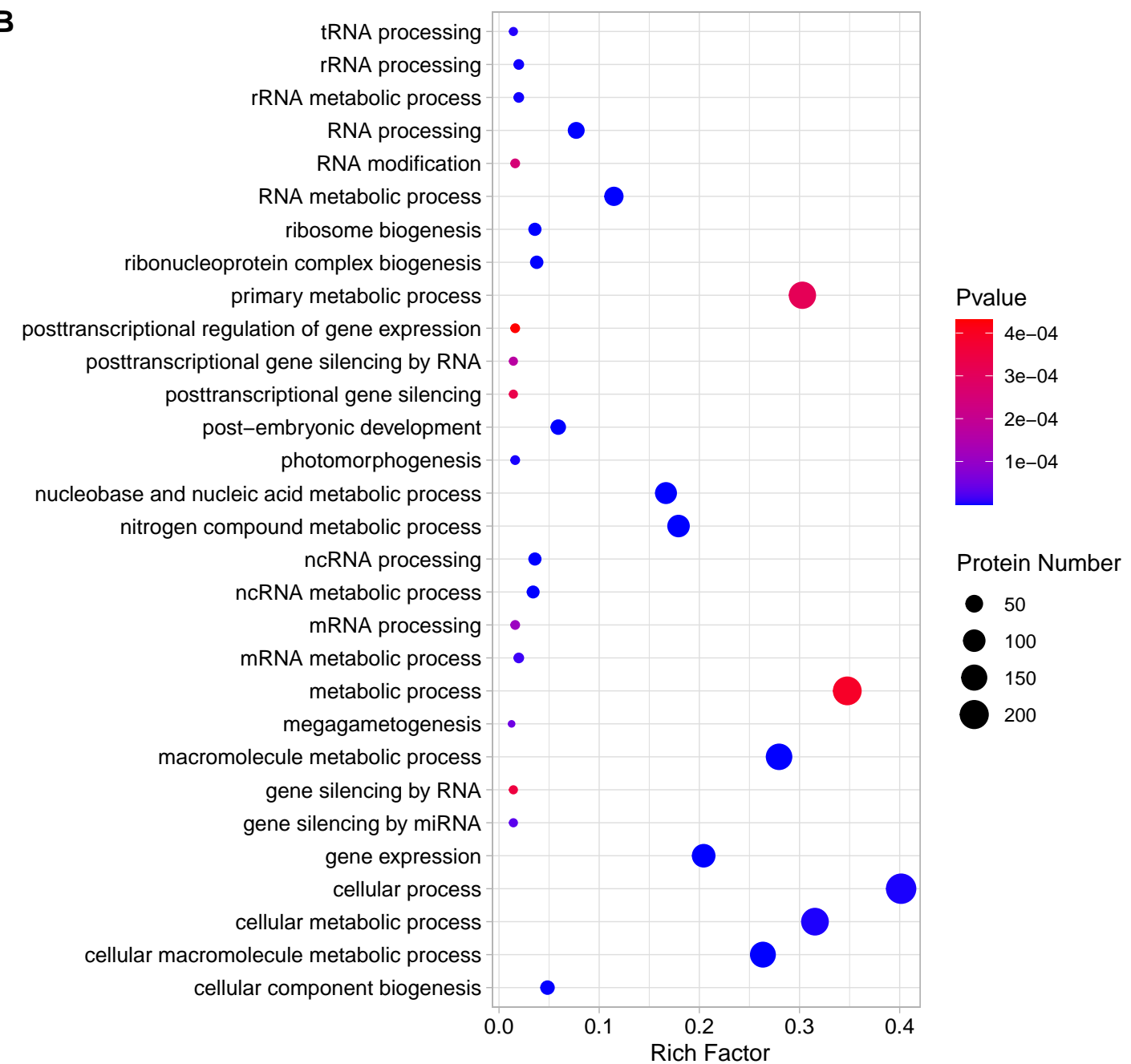

Supplement: Supplementary Figure 4 — GO Enrichment results for specific up-regulated genes in shoot tissues under heat treatment. (A) Enriched GO terms for shoot specific up-regulated genes under heat treatment at 1 h. (B) Enriched GO terms for shoot specific up-regulated genes under heat treatment at 5 h. [file Image_4.PDF]

A

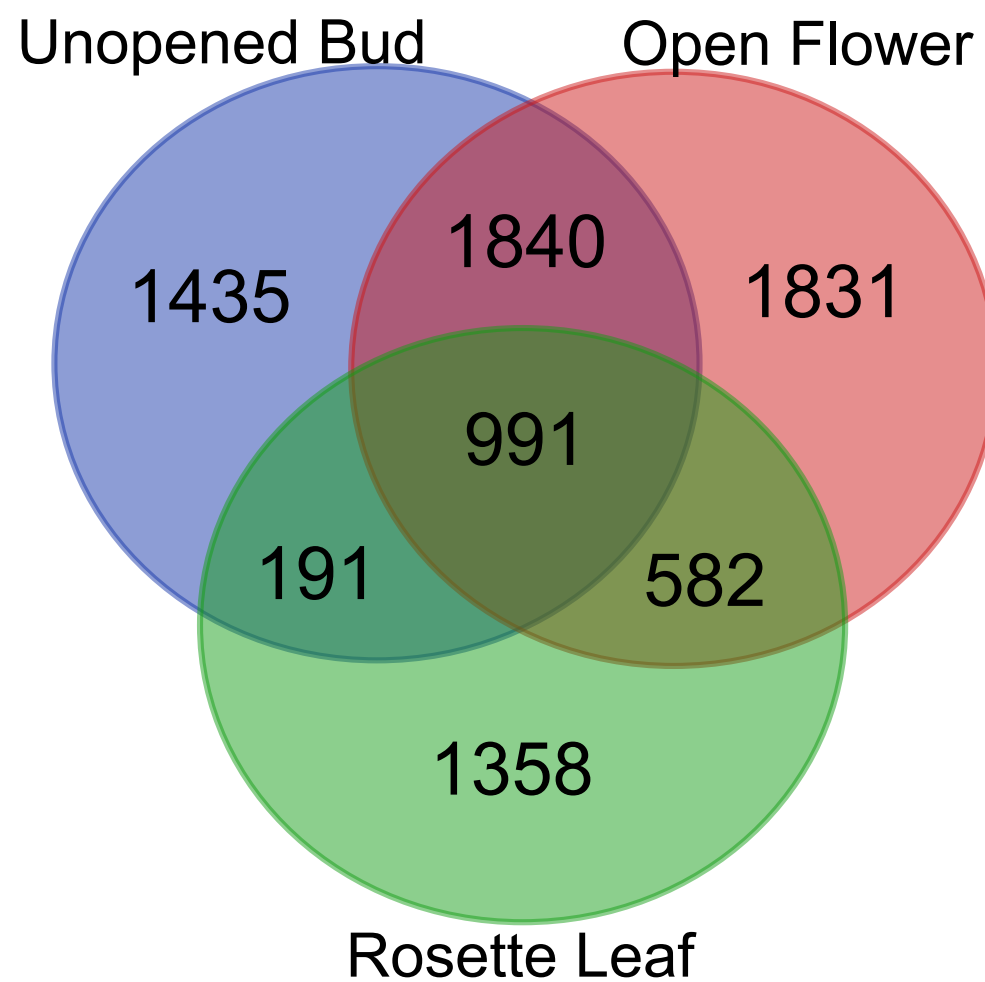

B

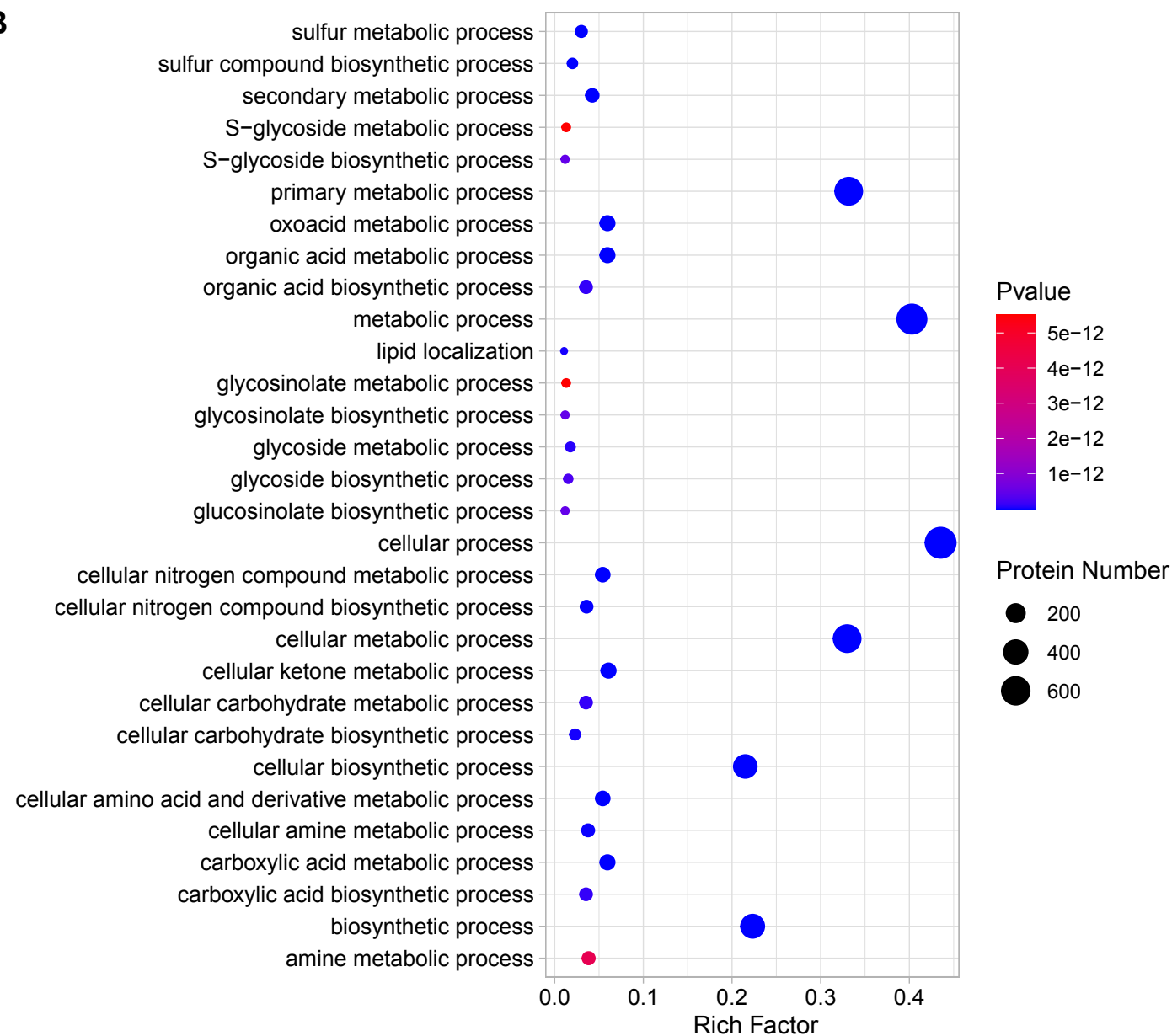

C

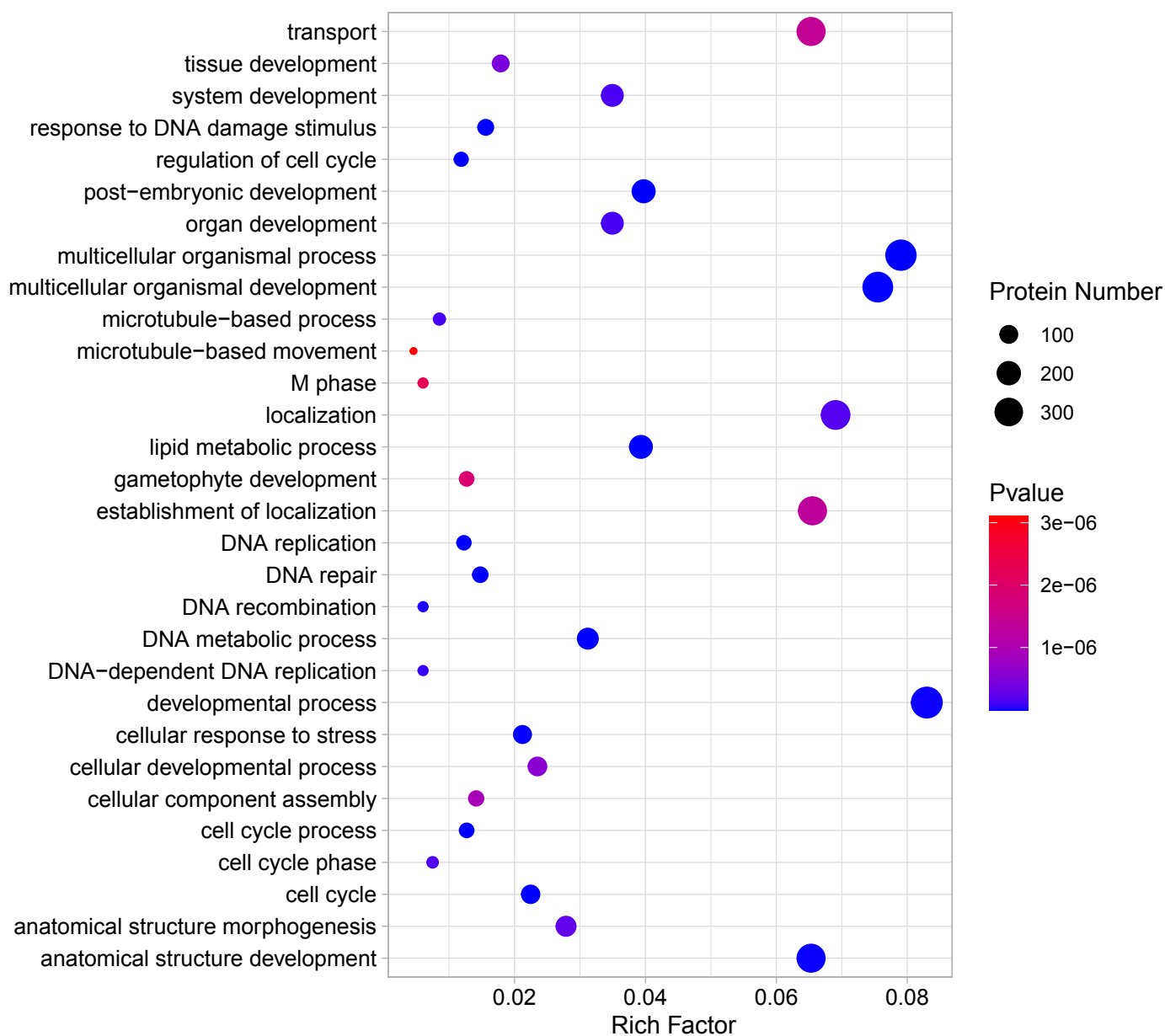

D

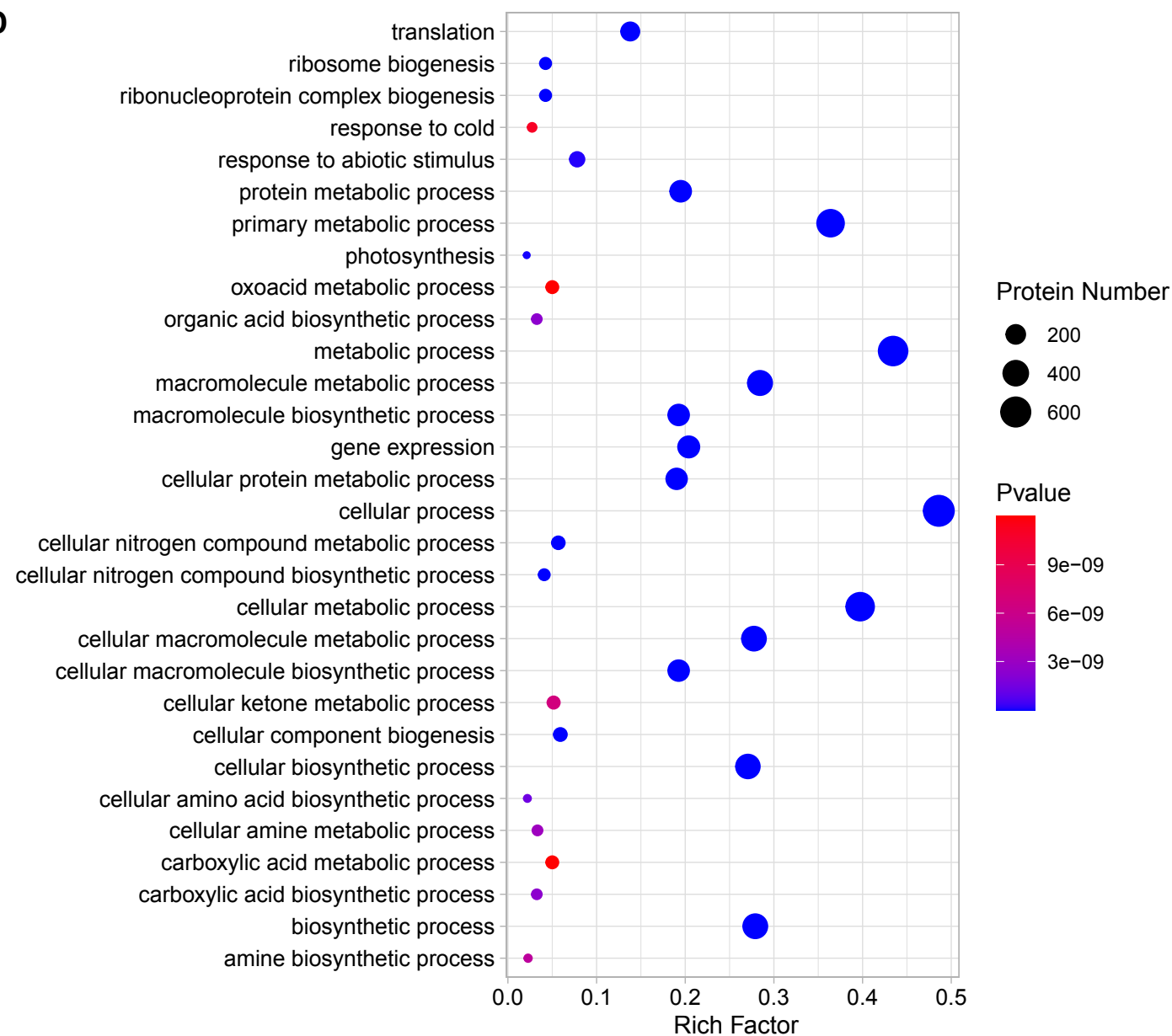

Supplement: Supplementary Figure 5 — GO Enrichment results for down-regulated genes between reproductive tissues (Bud and OF) and vegetative tissues (RL). (A) Venn graph for down-regulated genes between reproductive tissues (Bud and OF) and vegetative tissues (RL). (B) Enriched GO terms for commonly down-regulated genes between reproductive tissues (Bud and OF) and vegetative tissues (RL). (C) Enriched GO terms for specific down-regulated genes in reproductive tissues (Bud and OF). (D) Enriched GO terms for specific down-regulated genes in vegetative tissues (RL). [file Image_5.PDF]

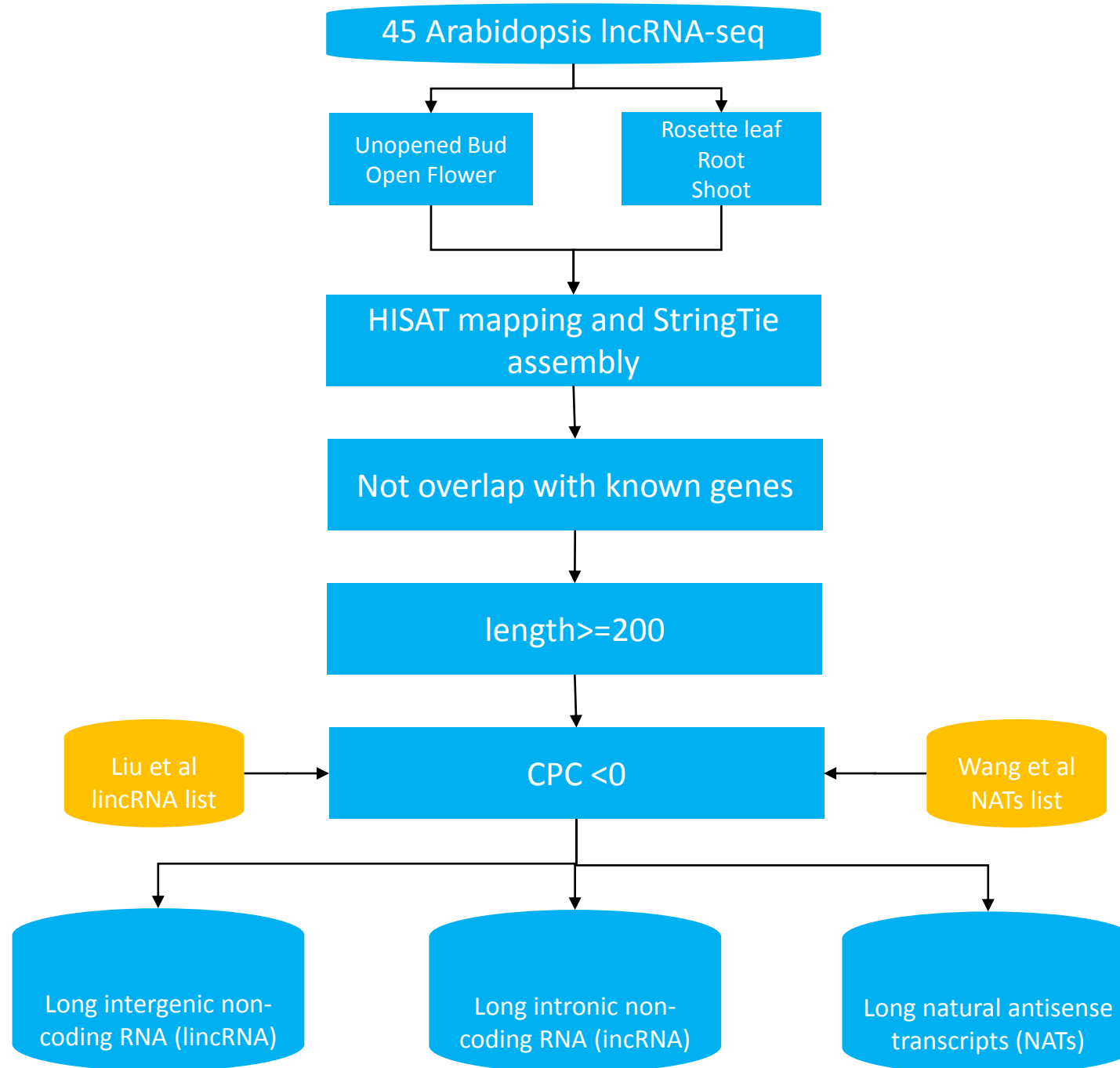

Supplement: Supplementary Figure 6 — Pipeline for lncRNA identification in Arabidopsis. [file Image_6.PDF]

**A**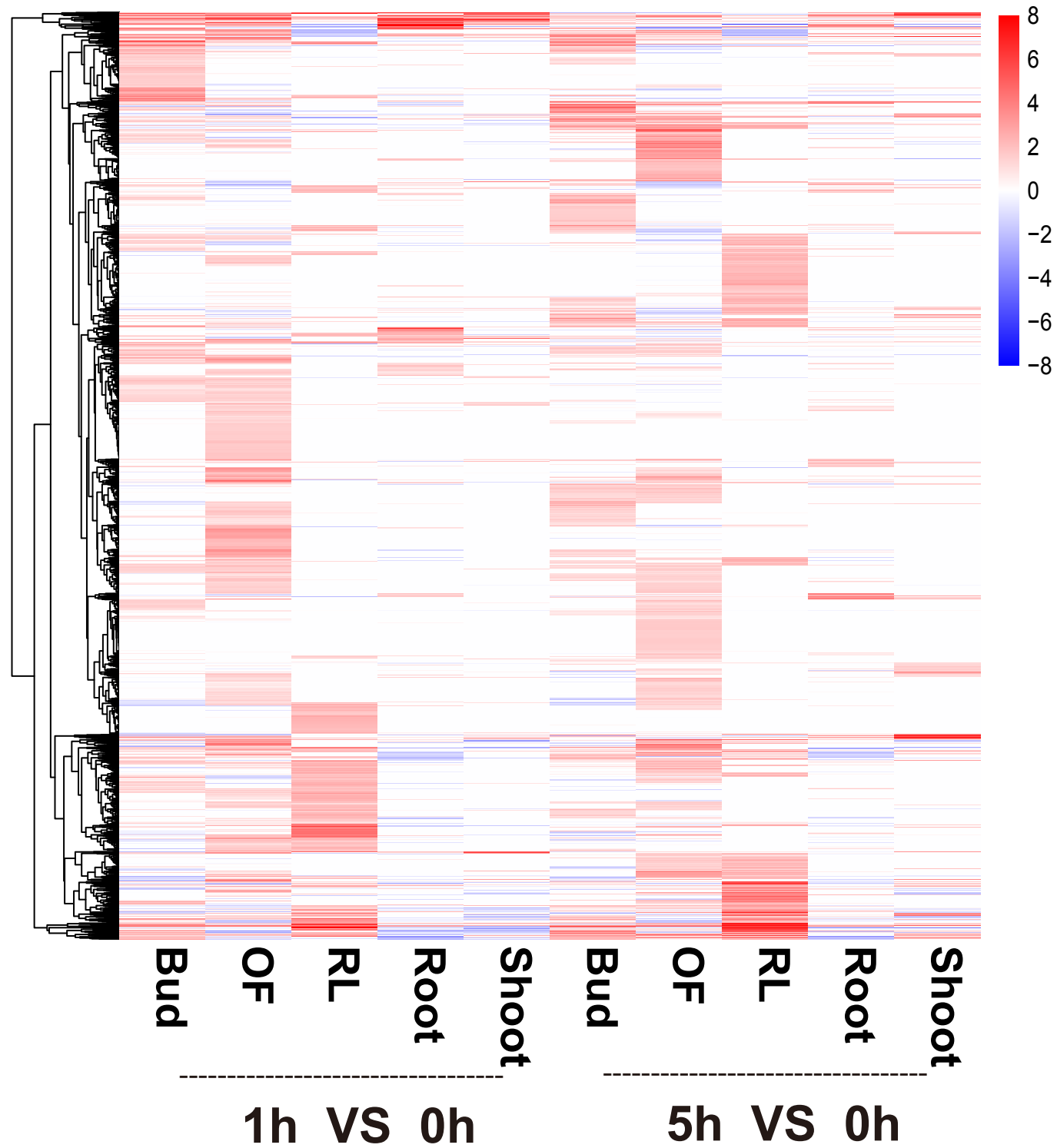**B**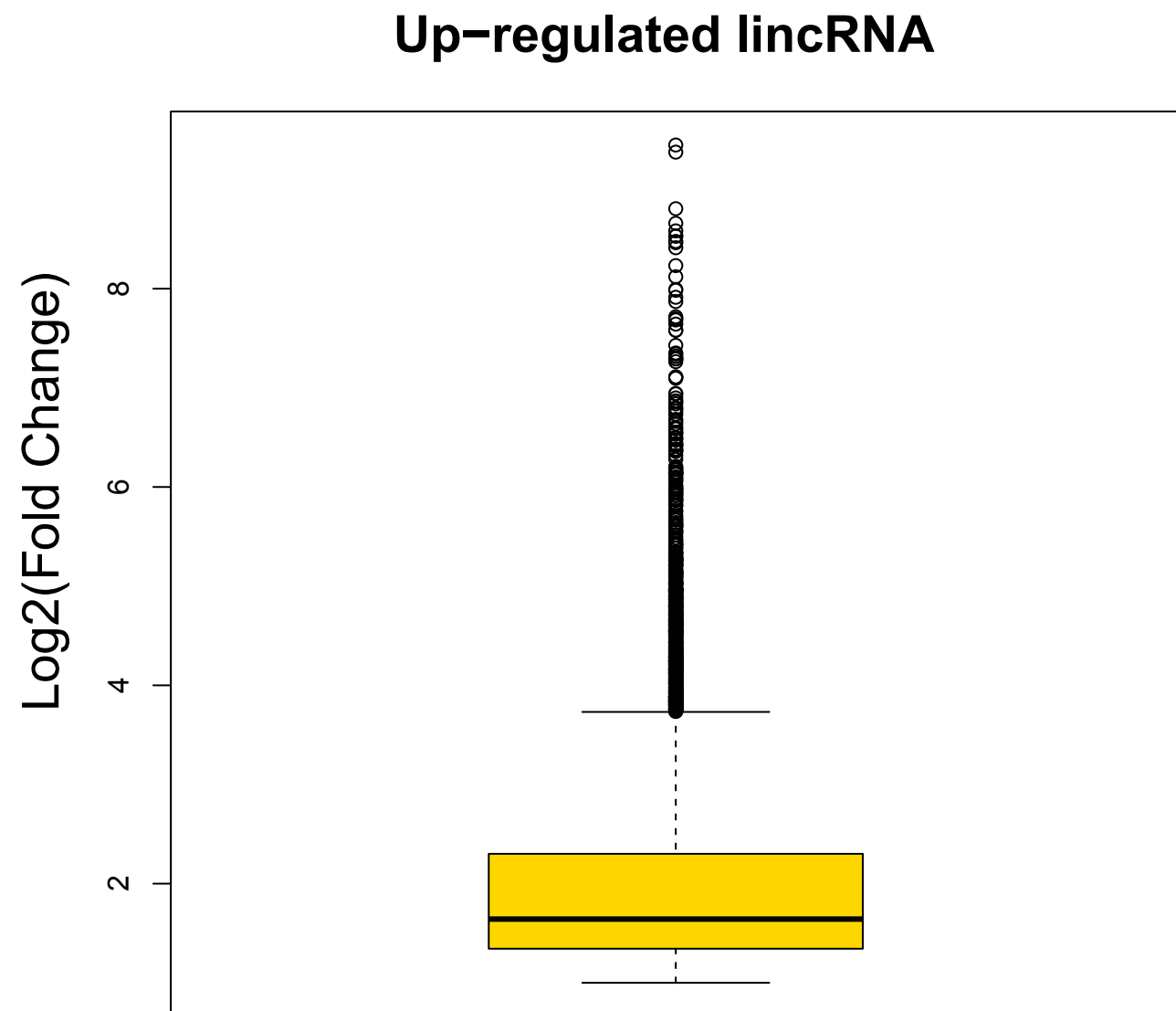

Supplement: Supplementary Figure 7 — Heatmap and boxplot for up-regulated lincRNAs under heat treatment. (A) Heatmap for up-regulated lincRNAs under heat treatment. X axis represents the log2 fold change (FC) in transcript abundance between control sample and heat treated sample. (B) Boxplot for up-regulated lincRNAs under heat treatment. [file Image_7.pdf]

A

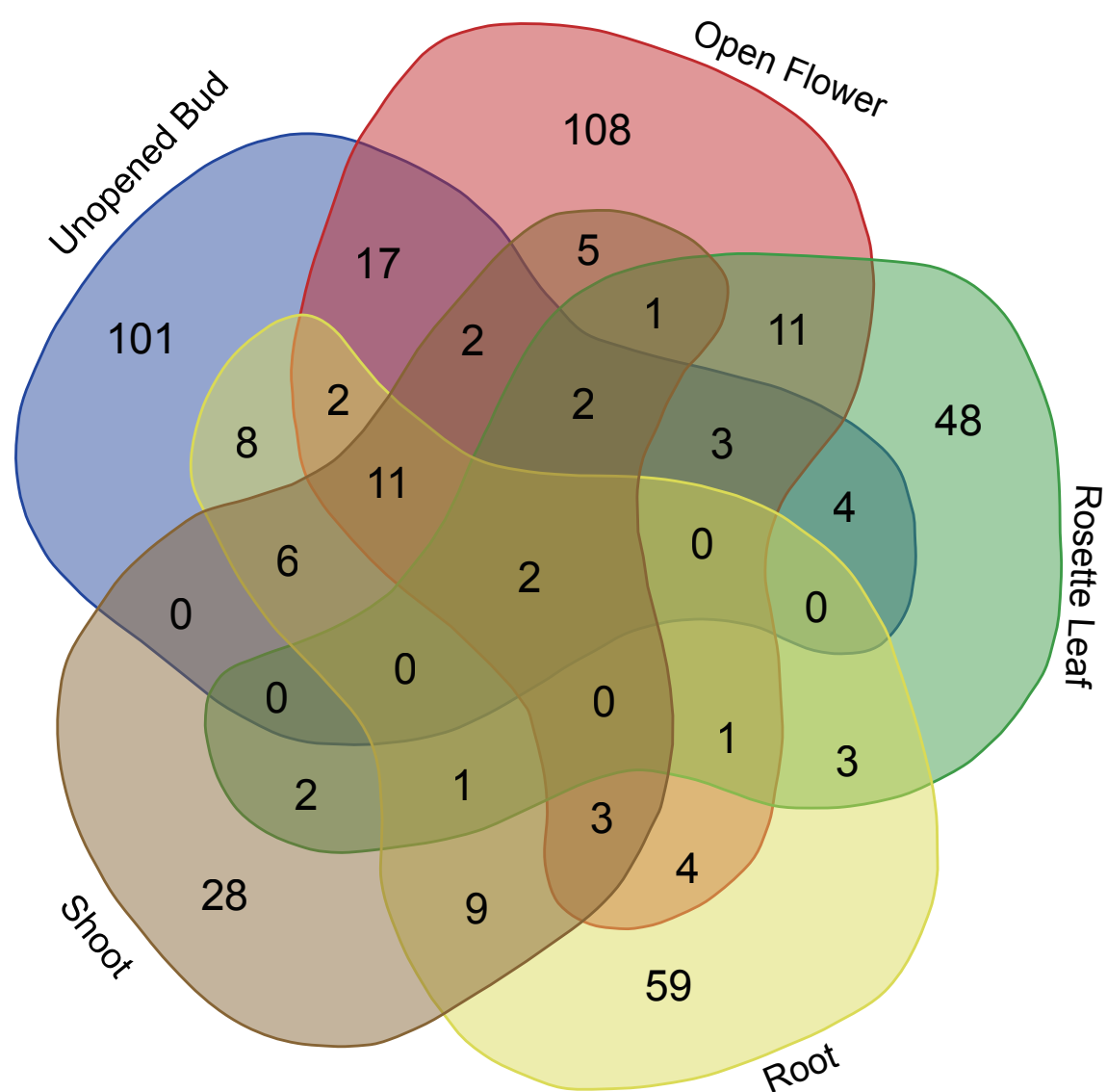

B

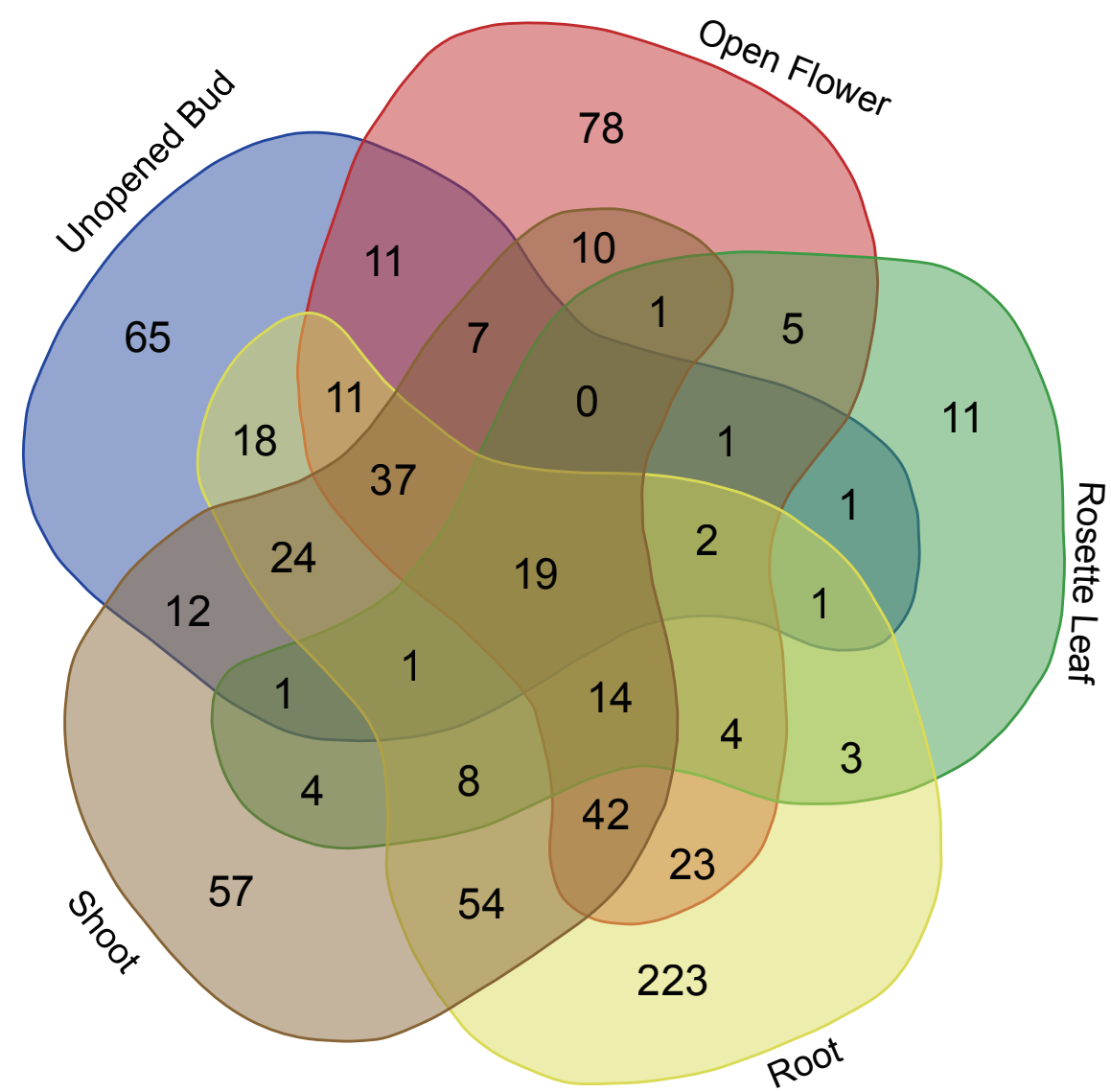

C

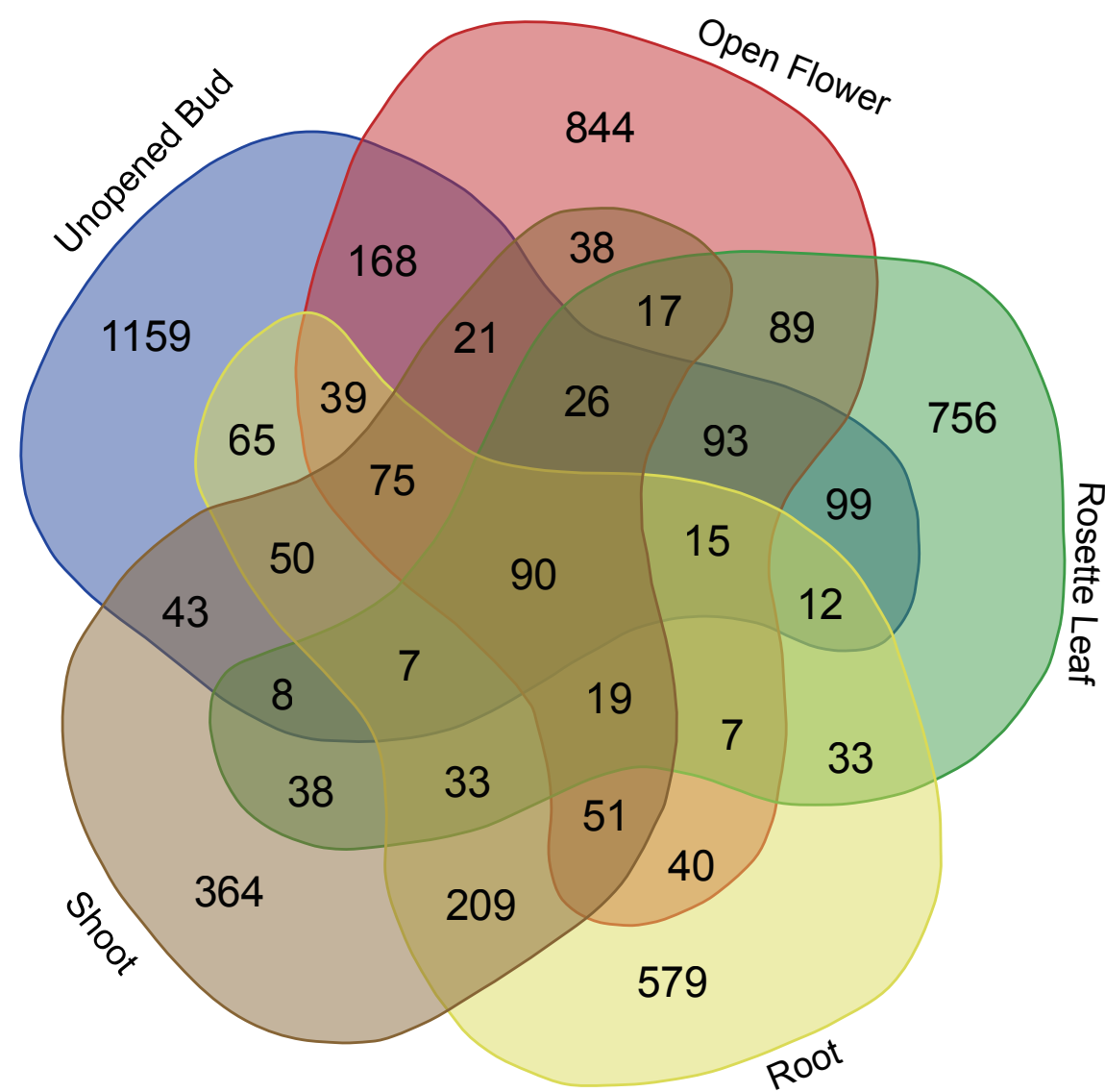

D

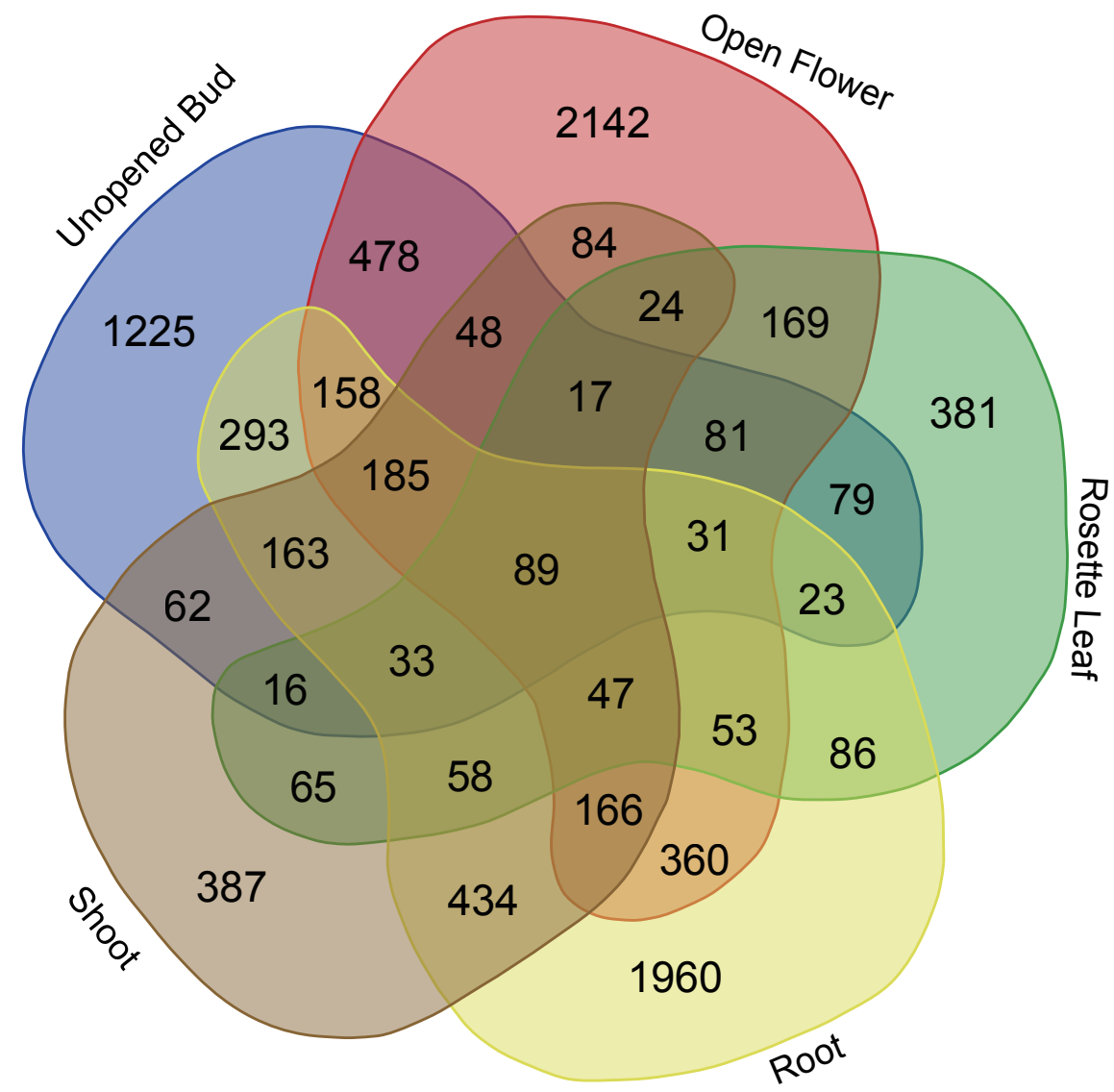

Supplement: Supplementary Figure 8 — Venn graph for differentially expressed lincRNAs and lncNATs under heat treatment. (A) Venn graph for up-regulated lincRNAs under heat treatment. (B) Venn graph for down-regulated lincRNAs under heat treatment. (C) Venn graph for up-regulated lncNATs under heat treatment. (D) Venn graph for down-regulated lncNATs under heat treatment. [file Image_8.PDF]

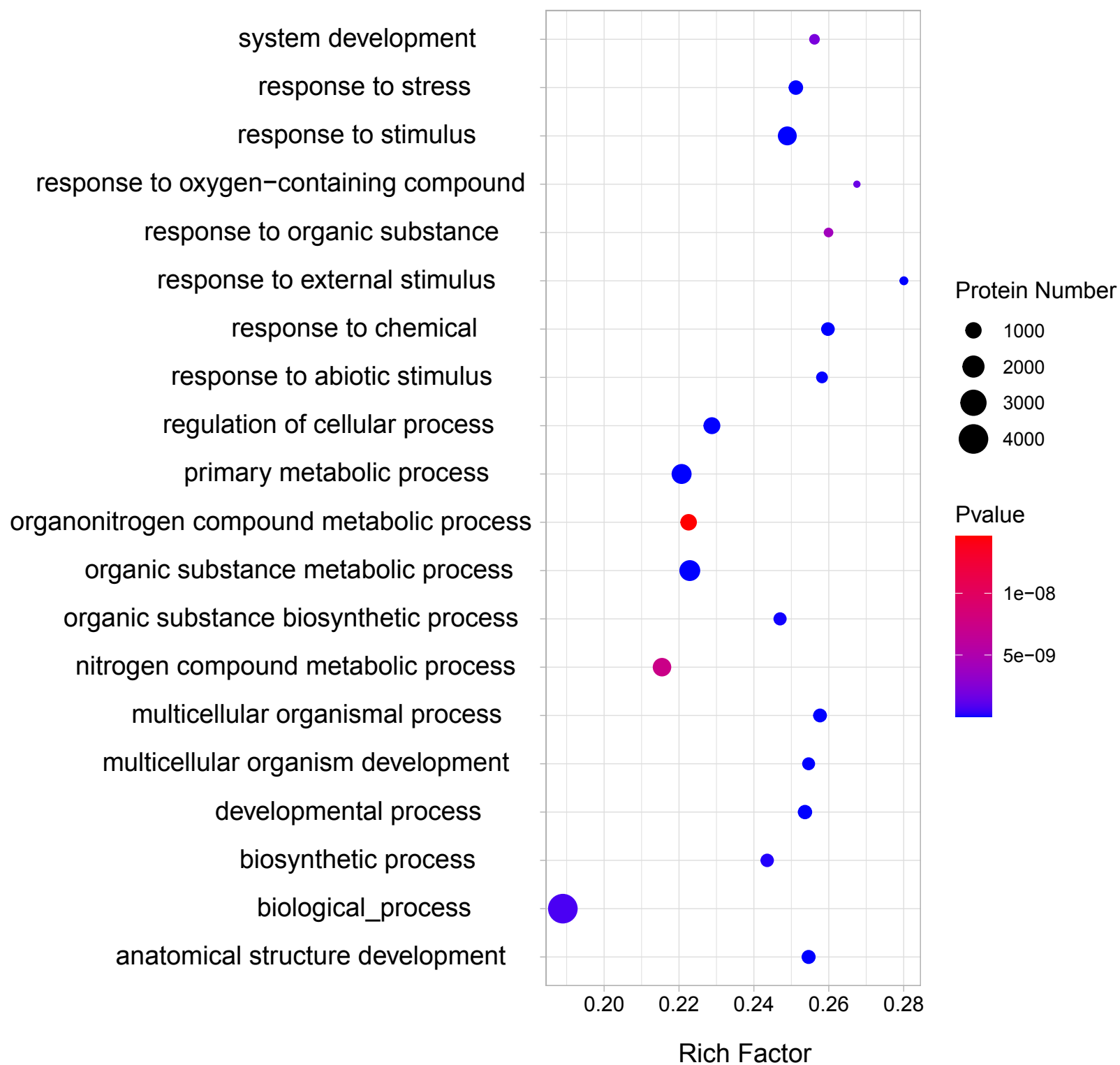

Supplement: Supplementary Figure 9 — Enrichment GO terms for neighboring genes of differentially expressed genes encoding lincRNAs under heat treatment. [file Image_9.PDF]

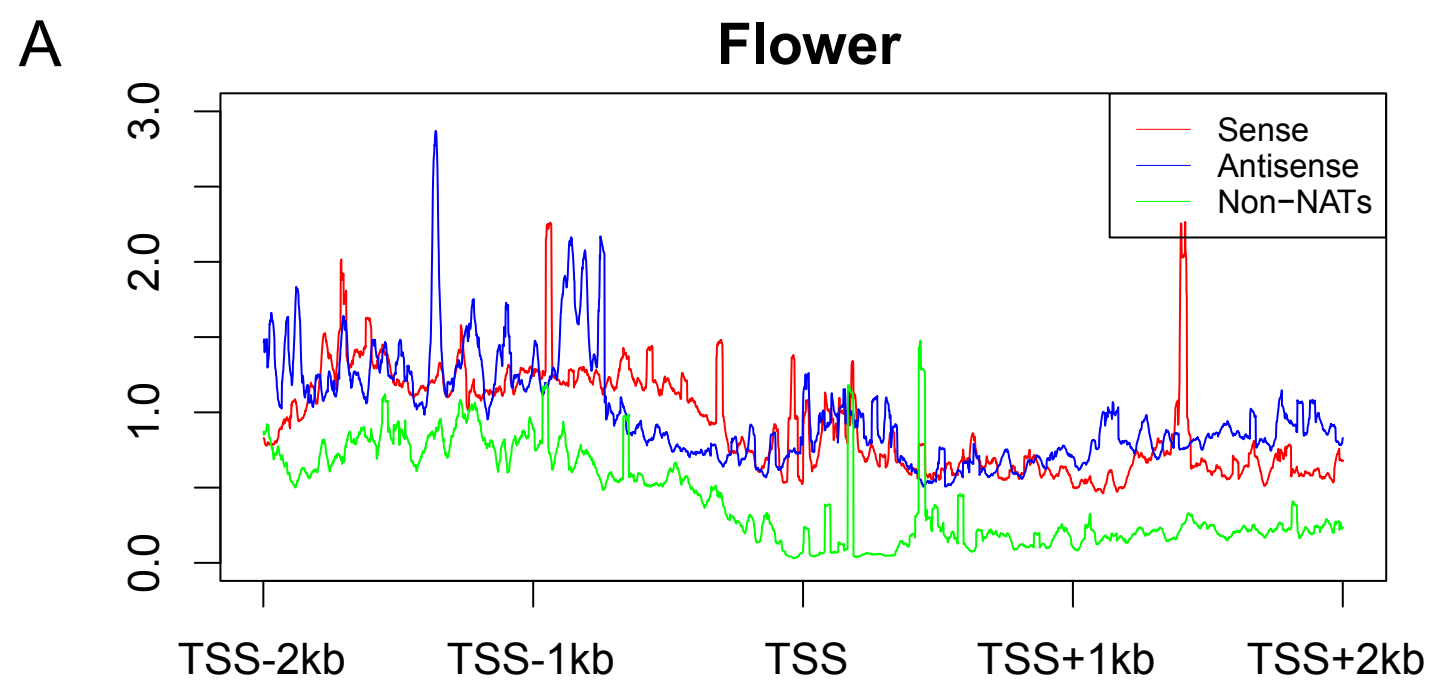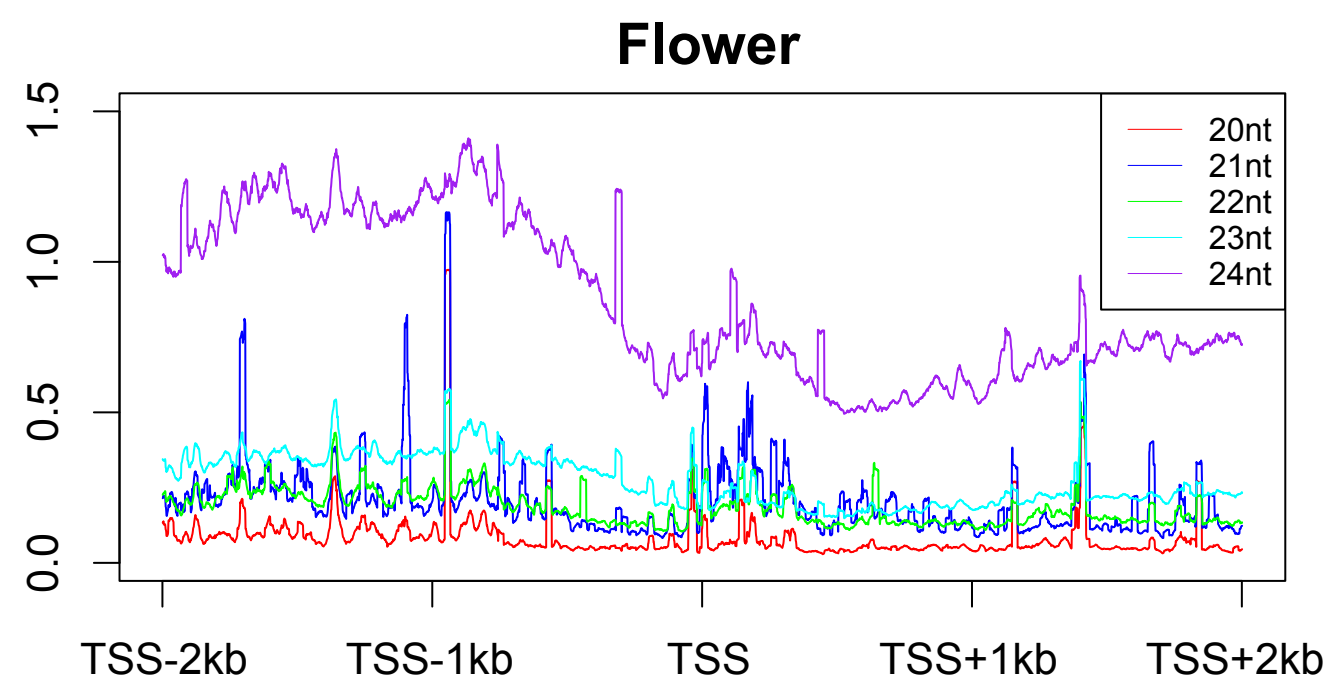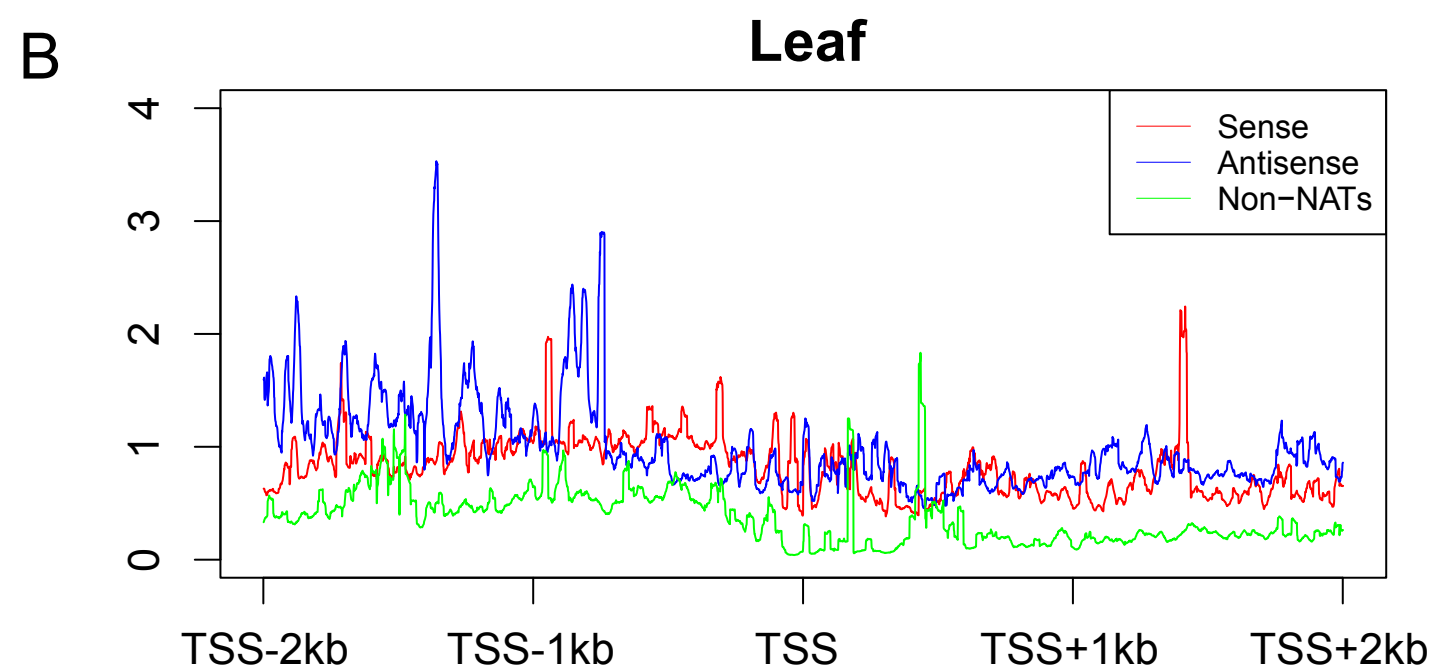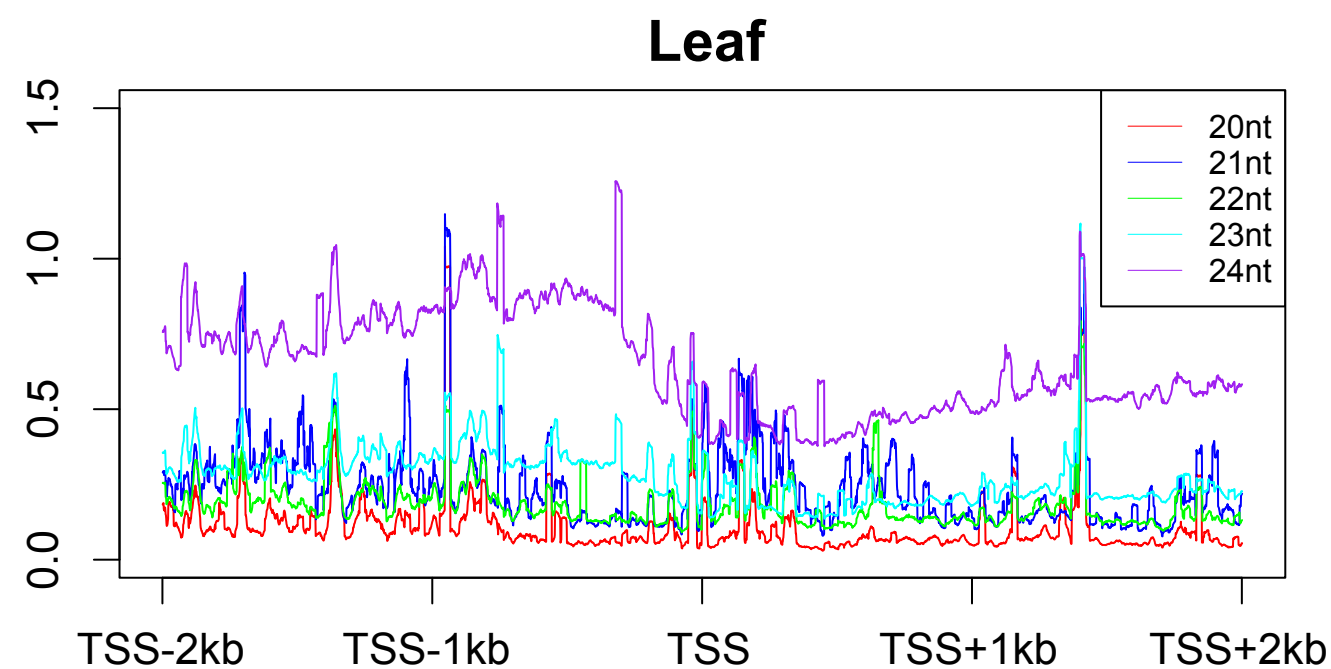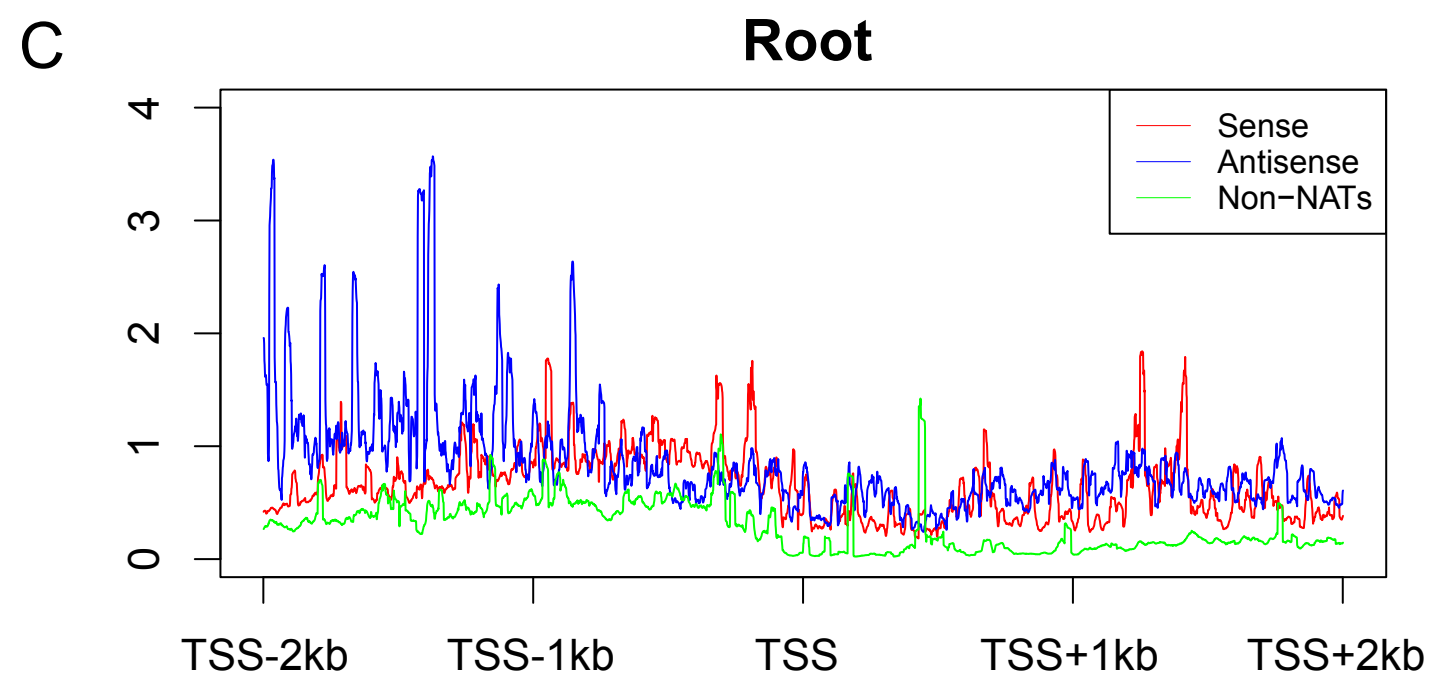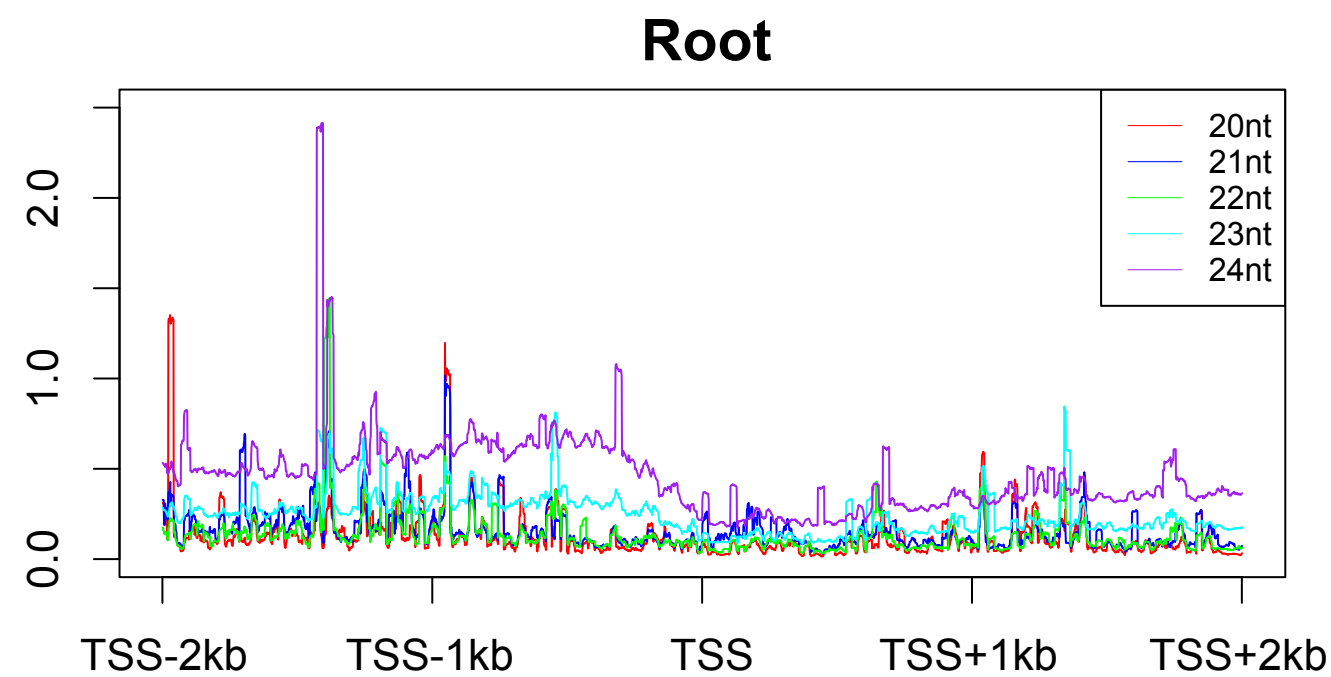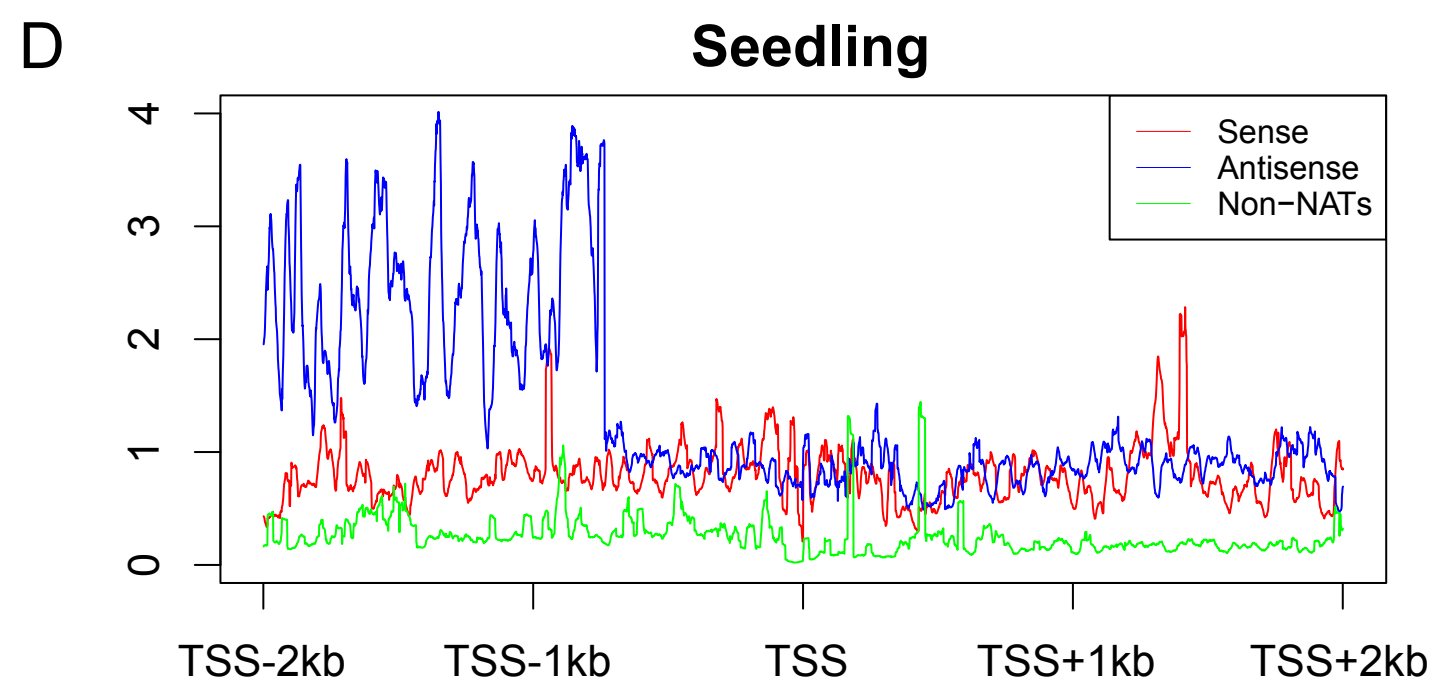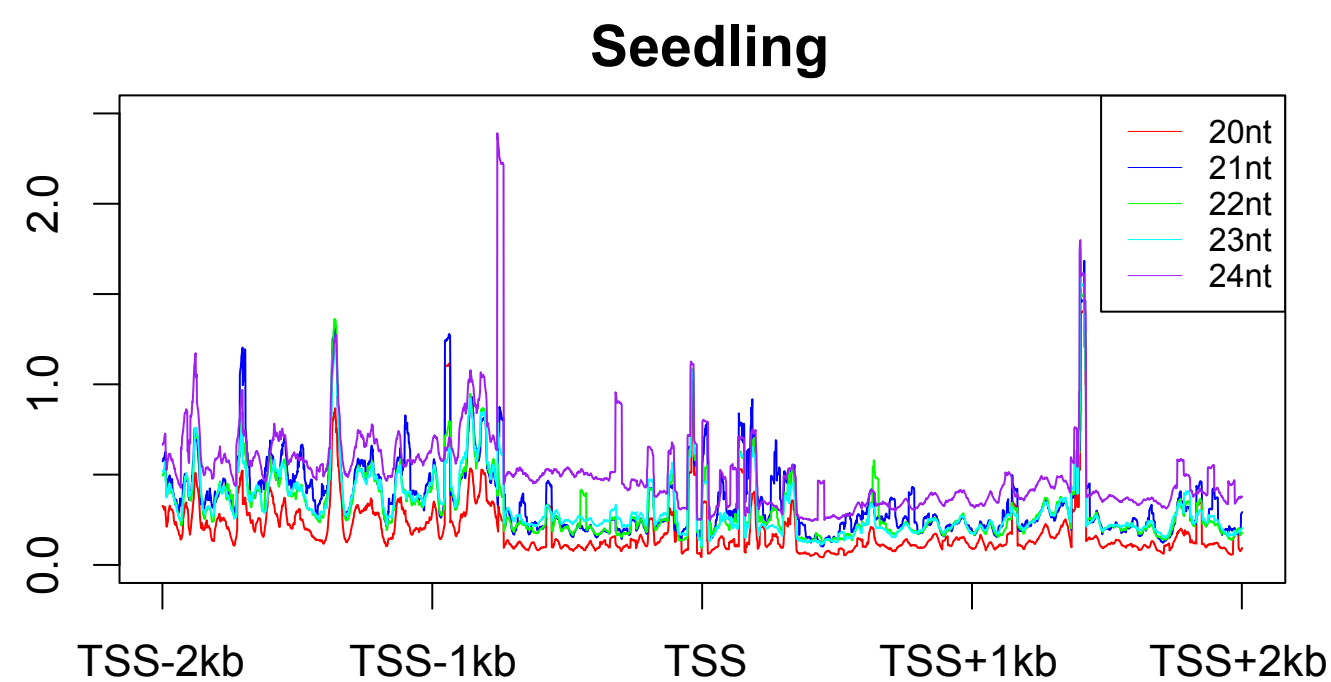

Supplement: Supplementary Figure 10 — Distribution of small RNAs in flower (A), leaf (B), root (C) and seedling (D) around transcription start sites (± 2000-bp) of sense and antisense genes of NATs and non-NAT genes. [file Image_10.PDF]
